# Predicted transcription factor binding sites as predictors of operons in *Escherichia coli *and *Streptomyces coelicolor*

**DOI:** 10.1186/1471-2164-9-79

**Published:** 2008-02-12

**Authors:** Emma Laing, Khushwant Sidhu, Simon J Hubbard

**Affiliations:** 1 Faculty of Life Sciences, The University of Manchester, Michael Smith Building, Oxford Road, Manchester, M13 9PT, UK; 2 School of Biomedical and Molecular Sciences, University of Surrey, Guildford, GU2 7XH, UK

## Abstract

**Background:**

As a polycistronic transcriptional unit of one or more adjacent genes, operons play a key role in regulation and function in prokaryotic biology, and a better understanding of how they are constituted and controlled is needed. Recent efforts have attempted to predict operonic status in sequenced genomes using a variety of techniques and data sources. To date, non-homology based operon prediction strategies have mainly used predicted promoters and terminators present at the extremities of transcriptional unit as predictors, with reasonable success. However, transcription factor binding sites (TFBSs), typically found upstream of the first gene in an operon, have not yet been evaluated.

**Results:**

Here we apply a method originally developed for the prediction of TFBSs in *Escherichia coli *that minimises the need for prior knowledge and tests its ability to predict operons in *E. coli *and the 'more complex', pharmaceutically important, *Streptomyces coelicolor*. We demonstrate that through building genome specific TFBS position-specific-weight-matrices (PSWMs) it is possible to predict operons in *E. coli *and *S. coelicolor *with 83% and 93% accuracy respectively, using only TFBS as delimiters of operons. Additionally, the 'palindromicity' of TFBS footprint data of *E. coli *is characterised.

**Conclusion:**

TFBS are proposed as novel independent features for use in prokaryotic operon prediction (whether alone or as part of a set of features) given their efficacy as operon predictors in *E. coli *and *S. coelicolor*. We also show that TFBS footprint data in E. coli generally contains inverted repeats with significantly (p < 0.05) greater palindromicity than random sequences. Consequently, the palindromicity of putative TFBSs predicted can also enhance operon predictions.

## Background

Genes within an operon are, in the majority of cases, transcribed from a single promoter [1]. Therefore, any integrated approach to the understanding of gene expression in prokaryotic systems must consider operons as one of the basic units, which are regulated by promoters, transcription factors and associated proteins. Although over 500 bacterial genomes have been sequenced and microarray-based expression studies are increasingly common, defining and mapping operon structure is time-consuming and complex, and the ability to predict them *ab initio *is therefore important. Since operon structure is frequently unstable across species boundaries [2-8], most approaches have used non-homology based data to predict operon structure. Besides predicted promoters and terminators, intergenic distance and expression data are also often used in non-homology based operon prediction strategies with varying sensitivity [9-17]. However, since there is generally limited data on known promoter locations and/or unreliable prediction methods, the identification of promoters is often difficult and makes their use problematic for operon prediction. In an attempt to avoid the problems associated with prediction of promoters it is worthwhile investigating the usefulness of other regulatory binding sites as potential predictors of operons, something not previously attempted.

Aside from promoters and terminators, transcription factor binding plays a key role in controlling the expression level of a gene or group of genes. Therefore, to coordinate the expression of genes within an operon a transcription factor binding site (TFBS), just like promoters, should be found predominantly upstream of the first gene in an operon; although there are a small number of reported cases of secondary binding sites within operons e.g. *lac *operon [18] and in our previous study [19], the traditional, 'static' view of operons (a group or genes transcribed together in all conditions) is applied. Prokaryotic transcription factors are usually deemed as either activators or repressors (with some having a dual role) depending on the effect they have on the rate of transcription initiation of the downstream gene(s). The dimeric nature of repressor and activator binding in prokaryotes requires that a recognition site must be a dyad, and typically an inverted repeat [20,21]. This 'structure' of a binding sequence coupled with their typically short length (6–15 bp) can be used to predict likely TFBSs themselves [21,22] and therefore if this can be done with confidence, can be used in the prediction of operonic gene pairs.

TFBSs are often described as consensus sequences or regular expressions that represent the most common sequence in a set of closely related binding site examples. Though simple in their construction, such consensus sequences are unable to imitate the variability that exists within true TFBSs in nature, where some sites may tolerate a range of bases and retain function. When using simple regular expressions, TFBS prediction is therefore dependent on the amount of mismatches (if any) that are tolerated. Defining the best consensus sequence for predicting sites is also difficult [23]. Position Specific Weight Matrices (PSWMs), first used for the characterisation of translation initiation sites [24] are an alternative to consensus sequences. The methods used to produce PSWMs differ mainly in the type of information used to collect a set of binding site examples. For example, the identification of regulatory motifs which can be used to predict regulons, commonly adopted after microarray data collection, uses a set of co-regulated genes as a prerequisite [c.f. [25,26]]. Similarly, prior knowledge can be used where experimental characterisation of a common regulatory binding site leads to the searching of upstream regions of the genes for a similar motif [27]. Knowledge of protein structure has also been applied to regulatory protein binding site identification through binding energy calculations between nucleotide and amino acid (e.g. [28,29]). In this study however, we wished to develop a method applicable to *Streptomyces coelicolor *genomics and due to the relative paucity of comprehensive information on *Streptomyces *gene regulation an *a priori *method that is capable of producing PSWMs is desirable. It will also support an operon prediction method that integrates data from various sources (e.g. intergenic distance [15] and/or expression data [14]) by increasing the amount of information about an operon whilst still being applied generically to all sequenced prokaryotic species without a dependency on the availability of functional or structural information.

One such method was published by the Siggia group [22] which requires no prior knowledge, can in principle be applied to any genome, and can generate PSWMs of dyads. The algorithm has successfully been applied in *E. coli *[22], *B. subtilis *[30] and additionally to *S. coelicolor *[31]. Here the published method [22] was implemented and applied in *Escherichia coli *and *Streptomyces coelicolor*.

By constructing PSWMs from over-represented words in a given data set it is possible to test the upstream regions of genes for putative TFBSs. Based on the 'classical' definition of an operon [1] intra-operonic genes (excluding the first gene of an operon) should not have a TFBS in their upstream region. Although this is increasingly viewed as overly simplistic as an operon definition [11,19,32,33] it appears to hold for the large majority of operons in most conditions. Subsequently, adjacent gene pairs that have a TFBS in between them are not expected to be part of the same operon. Using positive and negative examples of genes which are considered to be operonic members, the use of TFBSs as predictors of operons can be tested.

Prior to this, the first part of this work characterises the palindromic tendency of footprints of known transcription factors in *E. coli*, a property which is later shown to improve operon prediction in *S. coelicolor*. The second part of this work concentrates on developing a strategy of implementing the Siggia group's algorithm [22] through the use of different thresholds and examines the differences in results obtained. Comparisons with the previous TFBS set predicted in *S. coelicolor *[31] are also made. Finally the usefulness of TFBSs as predictors of operons in both *E. coli *and *S. coelicolor *is discussed.

## Methods

**Figure 1:**
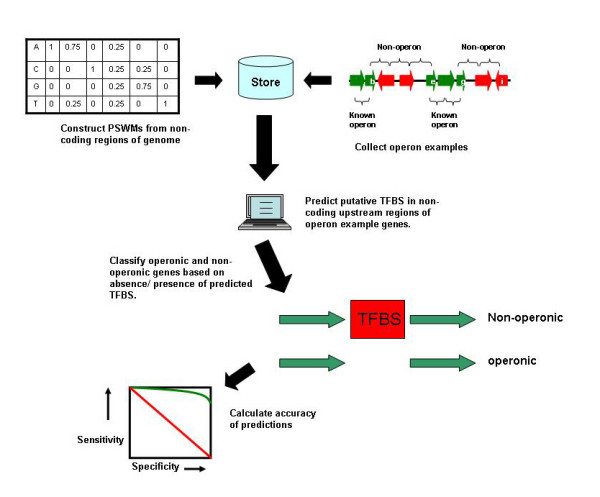
Overview of methods employed to measure operon prediction accuracy using Transcription Factor Binding Sites (TFBS).

Many methods are used to test the efficacy of TFBS at predicting operons in this work, which are summarised in Figure 1.

### PSWM construction algorithm

The algorithm published by Li and co-workers [22] is capable of taking any set of upstream sequences (referred to herein as UPSQs) and constructing PSWMs from significantly over-represented dimers within this set. This method can be summarised by five steps:

1) Counts of every possible dimer (*D*) of the form W_1 _N_*x *_W_2 _where W_1 _and W_2 _are short oligonucleotides of length 3–5 nt separated by *x *(0–30) arbitrary bases are collected from the UPSQs set. For each dimer a significance (-log_10 _P) is calculated by comparing the observed counts (*n*(*D*)) for the dimer to the expected counts (*y*(*D*)), assuming a Poisson distribution:

$$P=\sum_{n\geq n(D)}\frac{y^{n}(D)}{n!}e^{-y(D)}$$

where

$$y(D)=L_{eff}(D)\frac{n(W_{1})}{L_{eff}(W_{1})}\frac{n(W_{2})}{L_{eff}(W_{2})}$$

and *n*(*W*_1_) and *n*(*W*_2_) are the total number of occurrences of *W*_1 _and *W*_2 _in the UPSQs data set and $L_{eff}(M)=\sum_{r}\left(L(r)-L(M)+1\right)$ is the number of independent positions in the data where a motif *M *of length *L*(*M*) can be placed (*M *can be *W*_1_, *W*_2 _or *D*). The summation is over the regulatory regions (*r*) over all genes, each with a length *L*(*r*) (as taken from [22]).

2) A similarity matrix is constructed via an all-vs-all comparison of significant dimer pairs scoring above a designated threshold. Similarity between pairs of dimers is calculated by a simple scoring method, evaluating the number of matches minus the number of mismatches. Matches to N (an unknown base) or to any other base or overhangs are ignored (a heuristic method of sliding the sequences along to get the best score is applied). This score is then normalised to between 0 and 1.

3) The similarity matrix is used to cluster the significant dimers using CAST (Clustering Affinity Search Technique [34]) where a similarity threshold for clustering is set.

4) For every cluster, the actual sequences in the UPSQs set matched by any member of the cluster are extracted along with up to 10 flanking nucleotides (dependent on the position of the match and length of the UPSQ sequence). A multiple alignment of these sequences using CONSENSUS [35] produces an alignment matrix for that cluster.

5) Each alignment matrix is converted to a PSWM using the background base counts for the organism of interest. Here the background frequencies of A = 0.2953, C = 0.2059, G = 0.2023, T = 0.2965 were used for *E. coli *and A = 0.1589, C = 0.3494, G = 0.3420, T = 0.1497 for *S. coelicolor*.

A full description of the algorithm is given in the original publication [22].

In this work the thresholds used in steps 2 (dimer significance) and 3 (clustering threshold) are of the most interest. It is these thresholds that can affect the integrity of the PSWMs, which in turn could affect operon prediction accuracy. The different thresholds of dimer significance used were: -log_10 _P > 6, approximating to -log_10_(1/amount of all dimers tested for), -log_10 _P > 3, approximating to -log_10_(1/amount of all perfectly palindromic (i.e. palindromic without any mismatches) dimers tested for), and two intermediate thresholds of -log_10 _P > 4 and -log_10 _P > 5. Different clustering thresholds of 0.6, 0.7 and 0.8 were also tested.

### Prediction of TFBSs

With each PSWM there is an associated distribution of scores obtained when applied to a given data set of sequences. Two distributions of scores are obtained when scoring sequences from either the UPSQs set that were used to build the PSWM (collected in step 4 of the algorithm), or sequences from the UPSQs set that were not used to build the PSWM (sequences that will have a 'score' but should not have a significant match to the PSWM (as they were not used to build the PSWM) and thus provide a means of estimating noise in the PSWM prediction). These two Gaussian distributions, describe the 'real hits' and the background random matches respectively.

From the mean and standard deviation of the two distributions, the difference between the two distributions can be calculated. The larger the difference, the more specific the PSWM is deemed to be, which is formally represented as a Z-score:

$$Z=\frac{\mu_{real}-\mu_{background}}{\sigma_{background}}$$

Using the mean and standard deviation of the two distributions the specificity and sensitivity of searching for a potential TFBS can be controlled via two thresholds:

*threshold*1 = *μ*_*background *_+ *xσ*_*background*_

*threshold*2 = *μ*_*real *_- *xσ*_*real*_

where *x *represents any real number and affects the false-positive and false-negative rates when using *threshold1 *and *threshold2 *respectively.

In this study for a sequence to be predicted to contain a given TFBS the score of the sequence against the PSWM must be greater than μ_real_-2σ_real _(giving 98% coverage) and μ_background_+Sσ_background_, where *S *is used to represent trials of different thresholds from 0 to 10 in 0.5 increments as discussed further in this work. Since this term is the only part of the threshold that changes, the TFBS prediction threshold used will only be referred to herein as μ_background_+Sσ_background_.

### UPSQs data sets used

The sequence data used to form the UPSQs and operon prediction test sets were taken from *E. coli *K12 strain MG1655 from Genbank (accession number: NC_000913[36,37]) and *S. coelicolor *chromosome (not plasmids) in EMBL (accession number: AL645882 version 2 [38,39]). In *E. coli *the UPSQs set was created using the upstream intergenic sequences (maximum length 300 bp) of the first genes of known and predicted operons in the *E. coli *sequence taken from RegulonDB [40,41] as described previously [22]. For *S. coelicolor *upstream intergenic sequences (maximum length 300 bp) of all genes in the chromosome were used. Testing of the constructed PSWMs was conducted using the upstream intergenic sequences (maximum length 300 bp) of the positive and negative examples of operon members of the relevant organism.

### Positive and negative examples of operon members

**Figure 2:**
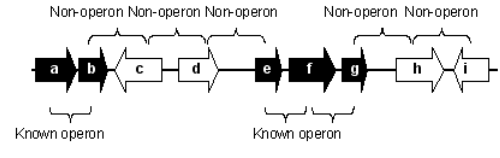
Examples of positive and control sets of operons/non-operons.

Operon definitions used throughout this work were based on the annotated genome of *E. coli *K12 strain MG1655 from Genbank [36,37] and the *S. coelicolor *chromosome (not plasmids) in EMBL [38,39], data sources described above. Positive examples of operons were collected through searching the literature for experimentally validated operons in *S. coelicolor *(e.g. [27]) and from the transcriptional unit experimentally based annotation in Ecocyc for *E. coli *[42,43]. Negative examples of operons were collected from knowledge of basic operon structure applicable to both organisms, illustrated in Figure 2.

Assuming that the entire polycistronic transcript is documented, non-operons of length 2 were formed by using the initial gene of the operon and its upstream neighbouring gene if that neighbour is transcribed in the same direction (e.g. gene pair d-e), and the last gene in the documented operon and its downstream neighbouring gene, again if it is transcribed in the same direction (e.g. gene pair g-h). These negative examples are termed as transcriptional boundaries. To increase the size of the non-operonic data set a non-operon of length 3 was also formed by collecting triplets of genes that are transcribed in the opposite direction (e.g. genes b-c-d), termed as directional examples, as the central gene (e.g. gene 'c' in Figure 2) is certainly not in the same transcriptional unit as the two flanking genes transcribed in the opposite direction (e.g. genes b and d in Figure 2). In total 35 operons and 1282 non-operons were collected for *S. coelicolor*, and 325 operons and 821 non-operons were collected for *E. coli*. Both positive and negative examples for *S. coelicolor *and *E. coli *are provided [see Additional file 1].

All examples of operonic genes (positive and negative) were split into singular genes such that the operonic connection status (in the same operon or not) of all genes with their respective upstream gene neighbours could be tested.

## Results

### Characterisation of inverted repeat binding sites

**Figure 3:**
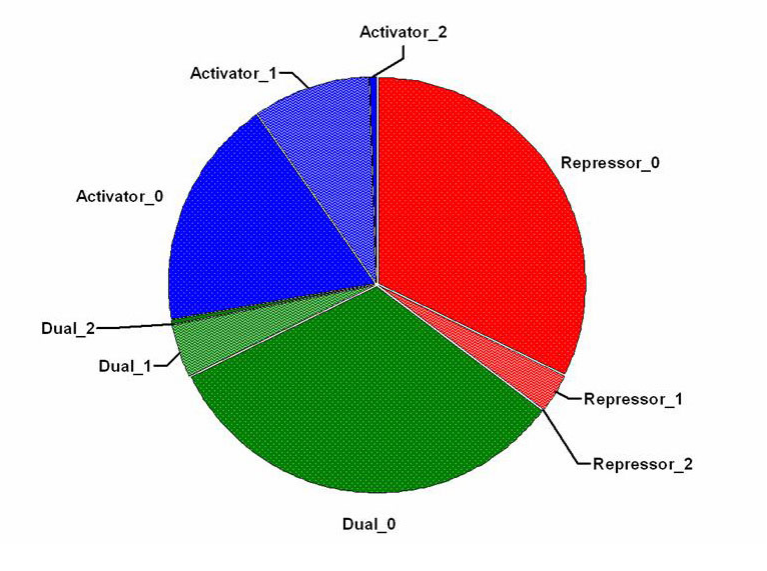
**Regulator type breakdown in TFBS footprints**. Pie chart of the percentage of footprints for each regulator containing an inverted repeat with both words greater than 3 nucleotides (includes palindromic matches of length 4 and 5) and palindromic mismatches between word 1 and word 2 of 0, 1 or 2. Data labels are of the form Regulator_mismatch.

Typically for prokaryotes the binding sites of repressors, dual regulators and in some cases activators are characterised as inverted repeats (where word 2 is palindromic to word 1), which is driven by the predominantly homo-dimeric binding of transcription factors [20]. However, 'perfect' palindromicity of a binding site where the two words are an exact match is not always the case e.g. the *lac *operator [44]. Given the tendency for binding sites to be imperfect palindromes it is therefore appropriate to analyse how many sites would be missed if only perfectly palindromic inverted repeat PSWMs were used when predicting TFBSs as a proxy for operons. Through the use of footprinting methods, where flanking nucleotides not bound by a transcription factor are degraded, a 'footprint' of the transcription factor can be obtained [45]. Although it is difficult to determine the precise binding site, the footprint sequences can be used as an estimate of the potential palindromicity of the experimentally characterised binding regions. Currently, there is little footprint data for *S. coelicolor *transcription factors and consequently transcription factor footprint data for *E. coli *was downloaded from the RegulonDB internet site [40,41], which contained examples of 211 repressor sites, 77 activator sites and 341 dual regulator sites [see Additional file 2]. All footprints for each set of regulators (Activators, Dual or Repressors) were then searched for the longest inverted repeat containing palindromes *word 1 *and *word 2 *and the 'palindromicity' calculated. Here, palindromicity was determined by the use of two thresholds; length and mismatches allowed. Figure 3 shows the percentage of known sites (be it repressor, activator or dual regulator) that can be characterised as an inverted repeat with each word of length 3, 4, or greater than 5 nucleotides, and having either 0, 1, or 2 internal palindromic mismatches between *word 1 *and *word 2 *(i.e. one cannot create an inverted repeat with words of length 4 by adding a mismatch onto perfectly palindromic words of length 3).

From Figure 3, and additionally Table 1, it is apparent that both repressors and dual regulators are predominantly characterised by perfectly palindromic dyads (84% and 79% respectively), agreeing with previous findings [46]. Activators on the other hand, described in the literature as capable of binding to direct/inverted repeats, are not (44%). Furthermore, the largest proportion of perfectly palindromic inverted repeats is formed from words of 3 nucleotides in length, particularly dual regulators (42%, data not shown). As expected, allowing for 1 internal mismatch increases the overall proportion of palindromic dyad sites matched compared to no mismatches allowed, but only a negligible increase is observed when considering 2 mismatches. Indeed, this typically just increased the length of the potential sites compared to the 1 mismatch set (data not shown).

**Table 1:** Coverage of inverted repeats in *E. coli *footprint data

Mismatches allowed	Random average coverage %	Footprint coverage %	Z-score	Associated p-value
Activators				
0	55	44	-2.4	0.008
1	82	74	-1.92	0.03
2	82	75	-2.54	0.006
Dual				
0	68	79	4.84	6.61E-07
1	96	96	1.54	0.06
2	96	97	0.09	0.46
Repressors				
0	66	84	6.43	0
1	90	91	0.36	0.36
2	91	91	-0.01	0.49
All regulators				
0	65	77	6.51	0
1	93	92	0.30	0.38
2	93	93	-1.09	0.14

Coverage of known sites and random sites (after 500 shuffle simulations) considering different mismatch tolerances is shown with associated Z-score and p-values for each regulator type. Coverage is defined as the percentage of sites (out of 341 dual regulators, 77 activators and 211 repressors) that are identified as being palindromic when allowing 0, 1 or 2 mismatches. The average coverage of 500 simulations is shown for the random data set (matched to the same number of examples for each respective TFBS set)

When considering all transcription factor footprints together, as depicted in Figure 3 and Table 1, the overall coverage obtained using palindromes and mismatches to characterise binding sites can be calculated. Coverage is defined as the percentage of all footprint sites identified as containing a palindrome. Here, 77% coverage can be achieved by searching for perfect palindromes and 92–93% coverage when allowing for 1 mismatch. However, it is important to note that tolerating mismatches decreases the specificity of binding site prediction. Of course, the occurrence of a palindromic inverted repeat within an upstream intergenic region does not necessarily mean that a sequence is a true binding site either, but it is expected to increase the likelihood. Indeed, in support of this, when comparing the coverage obtained (using Z-score) searching for perfect palindromes in true footprint sites to that obtained from 500 random footprint simulations a significant difference (p < 0.05) is observed (see Table 1). Here, random bases were chosen to create a sequence the same length of true, known footprint examples taken from those used to create Figure 3. No significant difference was observed when allowing 1 or 2 mismatches for Repressors and Dual regulators.

### Implementation of PSWM algorithm

Implementation of the PSWM construction algorithm using *E. coli *sequences resulted in 4,102 significant dimers using a Poisson probability threshold of -log_10 _P > 6. Although this is a similar threshold to previous work [22], these authors identified only 1,775 significant dimers. Despite rigorous checks with the implementation of this algorithm as published, and variation of parameters and similar experiments to reproduce the published data, this was not achieved and the implementation here consistently defined a larger number of putative sites. However, our implementation did discover all the dimers predicted by the earlier study [22], and this set was also used in our evaluation protocol.

Different thresholds of dimer significance (as described in the methods section) were then applied and sets of significant dimers were clustered using CAST [34] (step 3 of the algorithm) producing 12 data sets in total. In addition to these 12 sets of clustered sequences, the 849 clusters published previously [22], referred to as "LiFlank", were put through steps 4 and 5 of the PSWM construction algorithm. The resultant PSWMs for all data sets and the original PSWM data [22], referred to as "Li", were then used to predict binding sites in the upstream intergenic sequences of the positive and negative examples of operon members in *E. coli *with 0.5 increments of the TFBS prediction threshold μ_background_+Sσ_background _(see methods). For each increment of *S*, counts of TFBS predictions for the positive and negative tests sets were made enabling the calculation of true positives (TP), false positives (FP), true negatives (TN) and false negatives (FN).

Throughout this work we introduce a simple nomenclature to distinguish the PSWM sets, x_y_z, which refers to the Poisson distribution dimer significance threshold x, the word clustering threshold y, and the final number of clusters z.

**Figure 4:**
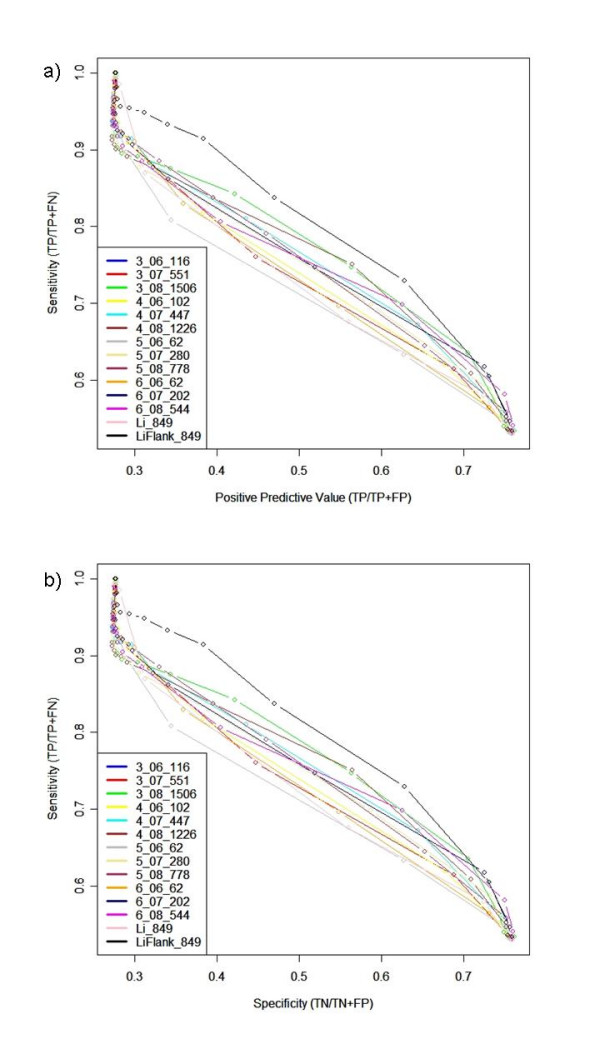
**Receiver Operator Characteristic curves for PSWMs as operon predictors**. ROC curves are shown for all 14 PSWM sets built and applied to *E. coli*. Legend refers to: Significance threshold or Data set_Clustering threshold_Number of clusters for a) Positive predictive Value (TP/(TP+FP)) vs sensitivity (TP/(TP+FN)), and b) Specificity (TN/(TN+FP)) vs sensitivity (TP/(TP+FN)).

Using predicted TFBSs as predictors of operons creates slightly unusual statistics, since an *absence *of an upstream predicted TFBS is predictive for a member gene of an operon. Hence when a high threshold is used, no TFBSs are predicted by the PSWMs leading to over-prediction of operonic status – i.e. all genes in both operon member test sets are classed as operonic. This leads to a sensitivity (coverage) of 1 and a specificity of 0, and the positive predictive value converges to the ratio of operonic/all genes. This is illustrated in the receiver operator characteristic (ROC) plots in Figures 4a and 4b. Similarly, at very low thresholds, TFBSs are over-predicted and most genes are classed as non-operonic. In this case, the sensitivity approaches 0 and the specificity approaches 1; the positive predictive value is undefined here. Figure 4a shows that at a low TFBS prediction threshold (where more TFBS are predicted) it is more likely that sequences without a predicted TFBS are operonic (although only reaching a positive predictive value peak of ~0%). As the TFBS prediction threshold increases, fewer TFBSs are predicted leading to less certainty about a gene's true operonic status. Indeed, only ~28% of genes predicted to be operonic are actually operonic at the highest TFBS prediction threshold. The predictions and ROC plots can be compared using a single metric, the area under the curve (A.U.C), which is a measure used to quantify the overall performance accuracy of predictions. The complete list of AUC values for different data sets is shown in Table 2. The best performing PSWM set at discriminating operonic/non-operonic genes is the LiFlank data set with 80% accuracy. This increase in accuracy compared to the original Li data set (76% accuracy) is likely due to the wider PSWMs which incorporate flanking sequence. During step 5 of the PSWM building pipeline CONSENSUS [34] requires a fixed pattern length to be supplied. In this work the length is determined by the length of the shortest sequence matched back to the cluster during step 4. Comparisons between the length of the LiFlank PSWMs and the original Li PSWMs sets revealed that the LiFlank PSWMs were predominantly wider. The wider PSWMs and the higher accuracy of the LiFLank data set therefore indicate that the length of the matrix is important for operon prediction. This is not surprising as the wider a PSWM is, the more specific it generally becomes.

**Table 2:** Area Under the Curve scores for PSWM data sets

Data set	A.U.C
3_08_1506	79%
3_07_551	77%
3_06_116	76%
4_08_1226	76%
4_07_447	78%
4_06_102	77%
5_08_778	77%
5_07_280	77%
5_06_62	75%
6_08_544	76%
6_07_202	76%
6_06_62	76%
Li_849	76%
LiFlank_849	80%

The AUC score is shown for each of the 14 PSWM sets when predicting operons using the positive and negative examples of operon members of *E. coli*. Data set nomenclature x_y_z refers to: x, Poisson distribution dimer significance threshold (-log_10 _P >) or Data set, y, Clustering threshold, and z, the number of clusters/PSWMs in data set (see also methods and text).

The second best operon predictor set (3_08_1506 with 79% accuracy) is of one of the novel PSWM sets constructed in this study (using all steps of the algorithm). The dimer significance of -log_10 _P > 3 and clustering threshold of 0.8 was therefore used to construct PSWMs for *S. coelicolor *resulting in 22,359 significant dimers producing 3,628 clusters/PSWMs (referred to herein as S3_08_3628). Previous work applying the same approach on *S. coelicolor *[31] reported 2,497 putative TFBSs. However, using dyad word lengths of 3–5 nt (used here) instead of just 4 nt results in additional matrices, as well as all of the matrices found previously [31].

### TFBSs for operon prediction

**Figure 5:**
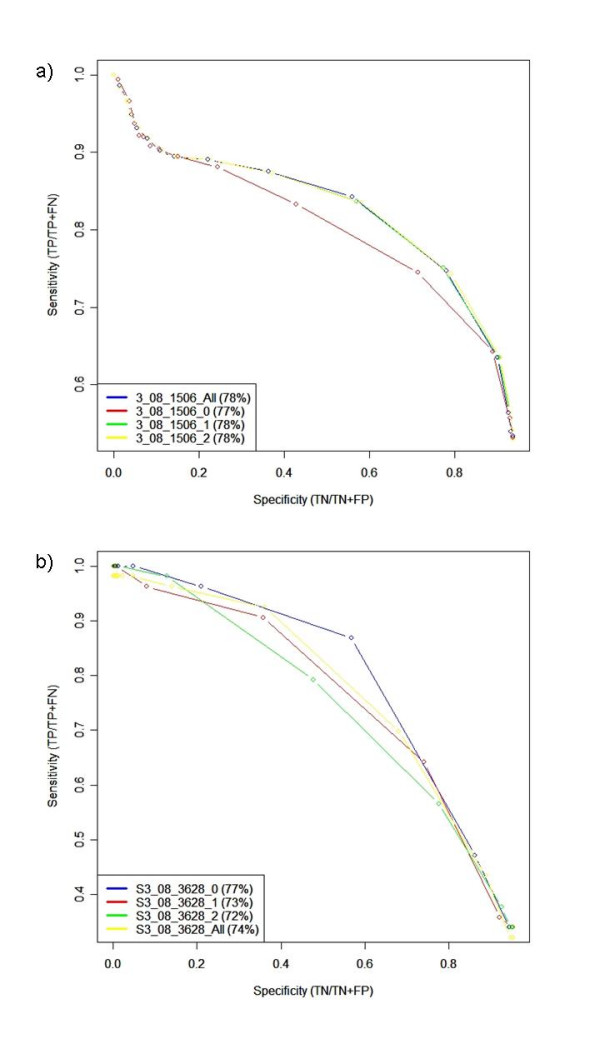
**Receiver Operator Characteristic curves of Specificity (TN/(TN+FP)) vs sensitivity (TP/(TP+FN))**. a) *E. coli *PSWM set 3_08_1506 and b) *S. coelicolor *PSWM set S3_08_3628; where legend refers to: Significance threshold or Data set_Clustering threshold_Number of clusters_mismatches allowed/all PSWMs (A.U.C).

Figures 5a and 5b show the ROC curves for *E. coli *PSWM set 3_08_1506 and *S. coelicolor *PSWM set S3_08_3628 respectively when applied to the relevant positive and negative operon member example datasets. When comparing AUC scores of the test sets using all of the PSWMs it can be seen that overall TFBSs perform better as predictors of operons in *E. coli *(79%) than *S. coelicolor *(74%). However, the AUC score of *S. coelicolor *increased to 77% when using only palindromic (no mismatches allowed) PSWMs (S3_08_3628_0, nomenclature of x_y_z_m referring to Poisson distribution dimer significance threshold x, the word clustering threshold y, final number of clusters z and the number of mismatches allowed m), calculated by using the consensus sequence conveyed by a PSWM [47] and applying the same palindromicity method as previously described. Conversely, operon prediction accuracy in *E. coli *did not improve when using only palindromic dimers of the original 3_08_1506 PSWM set.

**Figure 6:**
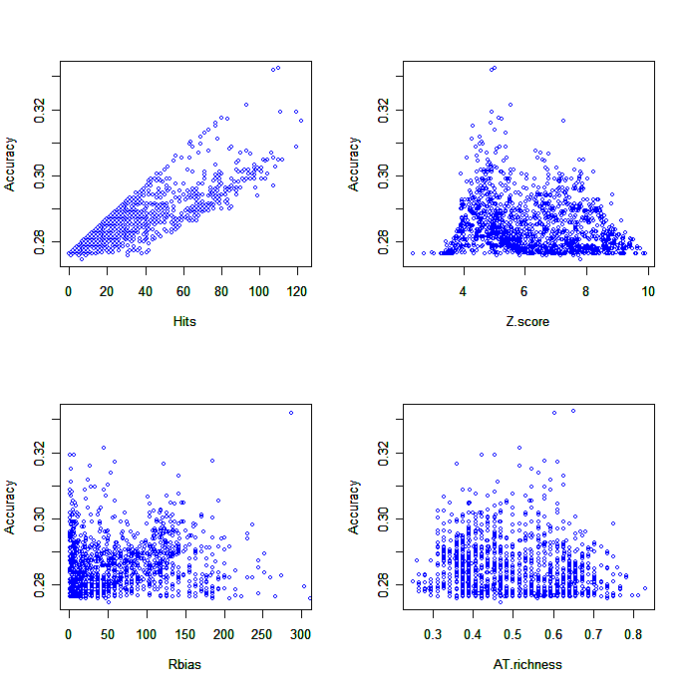
**PSWM statisitics for *S. coelicolor***. Z-score, number of hits, AT-richness and non-coding region bias (R_bias_) of each PSWM in the *S. coelicolor *S3_08_3628_0 PSWMs set using a TFBS prediction threshold of μ_background_+4σ_background_.

**Figure 7:**
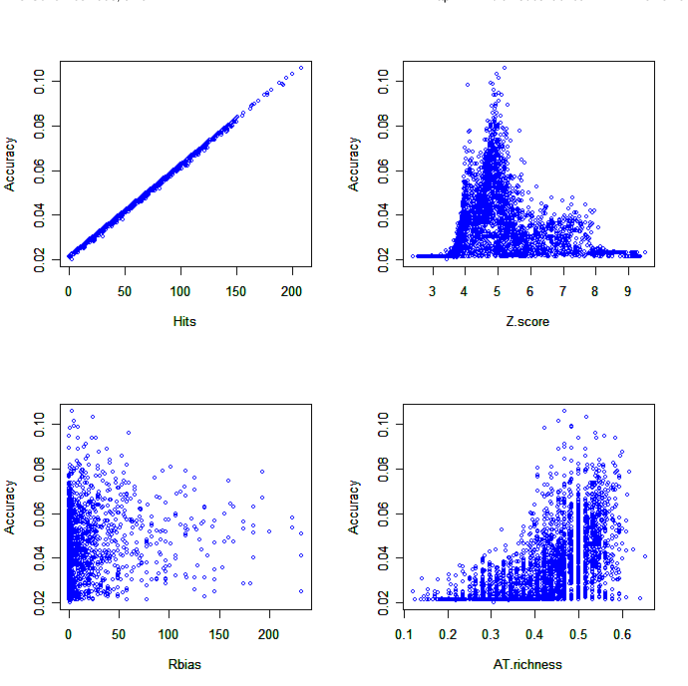
**PSWM statisitics for *E. coli***. Z-score, number of hits, AT-richness and non-coding region bias (Rbias) of each PSWM in the *E. coli *3_08_1506_all PSWMs set using a TFBS prediction threshold of μ_background_+4σ_background_.

The TFBS prediction threshold of μ_background_+4σ_background _with a very low false positive rate of ~0.003% was found to produce the highest operon prediction accuracy ((TP+TN)/(TP+FP+TN+FN)) in *E. coli *3_08_1506_all (83%) and *S. coelicolor *S3_08_3628_0 (93%). Using this threshold it was possible to analyse how a single PSWM's operon prediction accuracy is related to its Z-score (see methods), the amount of TFBS predictions it makes, its AT-richness (based on the consensus sequence), and its non-coding region bias (ratio of non-coding hits/coding hits when applied to the genome). The resultant plots are shown in Figure 6 for *S. coelicolor *and Figure 7 for *E. coli*.

From Figures 6 and 7 it is apparent, particularly in *S. coelicolor *(Figure 6), that the higher a PSWM's Z-score (and thus higher specificity), the lower the PSWM operon prediction accuracy. Somewhat counter intuitively, this suggests that the more successful PSWMs in terms of operon prediction accuracy predict many TFBSs (due to a low Z-score indicating lack of specificity in the prediction of an actual TFBS) such that very few operonic predictions are made. Further evidence for this can be seen in Figures 6 and 7 which show accuracy is correlated to the amount of hits, whereby many hits equates to higher accuracy. This demonstrates that high operon prediction accuracy is obtained by maximising sensitivity (coverage) of the TFBS prediction, albeit at the expense of TFBS prediction specificity, and without over-predicting TFBSs either.

Figures 6 and 7 also consider nucleotide biases. In *E. coli *the AT-richness of a PSWM does not appear to be related to accuracy due to the wide distribution of scores (Figure 7). In *S. coelicolor *however the matrices that are more AT rich tend to have a higher operon prediction accuracy, seen as a right shift in Figure 6. AT tracts in the transcription initiation region of genes are not uncommon [48] but when the PSWM operon prediction is restricted to using PSWMs of the set S3_08_3628_0 that have an AT-richness greater than 40%, no effect on operon prediction accuracy was found (data not shown). Finally there does not seem to be any relatedness between a PSWM's accuracy and its bias towards non coding regions in both *E. coli *and *S. coelicolor *(Figures 6 and 7 respectively).

## Discussion

To date non-homology based operon prediction methods that search upstream regions for motifs have only included promoters and/or terminators [9-12,17]. Data is presented here that uses a different upstream motif, transcription factor binding sites, to predict operons in both *S. coelicolor *and *E. coli*. Avoiding the need for experimentally validated examples of promoters/terminators and therefore dependency on the amount of prior knowledge available [9,10,12,17] this novel approach is able to predict operons in *E. coli *and *S. coelicolor *with 83% and 93% accuracy respectively. This strongly suggests that predicted TFBSs are a highly suitable feature for inclusion in integrated operon prediction strategies, not only in well characterised systems but also in other poorly-annotated but sequenced prokaryotes, where good quality TFBS predictions can be obtained *ab initio *with the protocol described here. It should be noted here that our definition of non-operonic genes is expanded by considering triplets with genes transcribed in opposite directions and that the overall prediction accuracy falls to around 64% when only the transcriptional boundary set are used. However, this drop off also occurs using intergenic distance as the sole predictor, reducing to 62% accuracy with the same data set (and same threshold as that optimised for the negative set including directional examples). Thus, our claim is not that predicted TFBS alone are superior operon predictors, rather that they should be used as independent features that offer a complementary approach.

Indeed, TFBS may offer other advantages. Thus far intergenic distance has proven to be the most accurate single feature for operon prediction in *E. coli *and *B. subtilis *[9,10,12-14,49]. Intergenic distance is implicitly linked to our method since TFBS identification/prediction is focused on intergenic non-coding regions only. Comparing the operon prediction accuracy in *S. coelicolor *of the TFBS and intergenic distance approach (using the same log-likelihood method documented in [15]) both features achieve similar accuracy (93–95%) when applied to the same positive and negative (both directional and transcriptional boundary) operon example data set. The operon predictions themselves however are not perfectly correlated between the two approaches, although there is a strong correlation – around 0.7 depending on thresholds used for TFBS and intergenic distance predictions. Hence, the two features are complementary, and their combination is expected to produce further improvement in operon prediction. Finally, although both methods perform equally well, TFBSs as predictors of operons are advantageous since additional regulatory information about a particular putative operon may be inferred.

This protocol derives genome-specific PSWMs (Position Specific Weight Matrices) representing significant dimers, which can subsequently be exploited for operon prediction. The majority (77% – 93%) of experimentally validated *E. coli *transcription factor binding footprints can be characterised as palindromic (either with or without mismatches allowed) and consequently the PSWM set was filtered prior to operon prediction. Restricting the PSWM data set to 'perfect' palindromes (no mismatches allowed) increased operon prediction accuracy in *S. coelicolor *but not *E. coli *and therefore, and supports the conclusion that genome-specific models for operon prediction should be employed [19,50]. The improved performance in *S. coelicolor *compared to *E. coli *may be a result of the PSWM construction strategy. The complete upstream intergenic regions were used in *S. coelicolor *whilst a restricted set based on annotated TFBSs was used in *E. coli *to find over-represented dimers. This would be expected to lead to increased coverage in *S. coelicolor*, and perhaps increased specificity in *E. coli*. This is borne out by the results, where high TFBS prediction coverage leads to superior operon prediction performance.

The false negatives (FNs) in this operon prediction strategy are intra-operonic genes (genes known to be transcribed together with its respective upstream gene) that have a predicted binding site (given the set of predicted PSWMs and applied threshold) in their upstream non-coding regions. The existence of these is not wholly surprising given our previous results [19] which demonstrated that intra-operonic genes with increased expression relative to the first gene of the same operon are more likely to have a predicted TFBS in their upstream non-coding regions. This intra-operonic promotion of gene expression has been noted by other groups [11,32,33]. Using a μ_background_+4σ_background _as a PSWM threshold we found that 23% of the *S. coelicolor *and 43% of the *E. coli *false negatives (35 and 227 FNs respectively) had higher expression than the first gene of the same operon. Therefore, when microarray expression data is available, it is possible to combine this with high quality TFBS predictions to improve the overall accuracy of operon prediction and gain information on the dynamic nature of some operons (specific, isolated control within operons for specific responses). This would not be possible with the application of intergenic distance alone to predict operons, and offers a way forward to address the true complexity of operon regulation.

## Conclusion

This work has demonstrated the suitability of predicted TFBSs as operon predictors independently. We are currently working on a genome specific operon predictor for both *S. coelicolor *and *E. coli *that is able to combine TFBSs and other features found to be useful in operon predictions (Laing *et al*, Manuscript in preparation). Integrated approaches to operon prediction in prokaryotes are clearly needed, since expression of genes within operons is affected by several factors, not least the presence of suitable transcription factor binding sites, and array-based data on its own is insufficient to understand this phenomenon. Indeed, a recently developed operon prediction method for *S. coelicolor *[11] that combines intergenic distance, expression data and terminators, whilst able to show comparable operon prediction accuracy to that presented here, is unable to predict 'dynamic' operons, defined as the isolated expression of a gene(s) from their 'common' (under 'normal' conditions) transcriptional unit to which they reside. The findings of this work suggests that TFBS are a useful feature for predicting operons of all prokaryotes in their own right but also have the capacity to allow the modelling of dynamic operon structures of an entire genome in a particular, specific condition (e.g. response to a stress) when combined with expression data [19].

## Authors' contributions

EL carried out all of the calculations, data processing and analyses, KS helped build datasets, and SJH designed experiments, drafted the manuscript and led the overall project. EL and SJH jointly conceived the overall project and revised the manuscript. All authors have read and approved the final manuscript written by SJH.

## Supplementary Material

Additional file 1 Operon definitions used in this study. This file contains list of operonic and non-operonic genes used in the datasets tested in this study, for Streptomyces coelicolor and E. coli. Data sets are divided into known operons, non-operon gene pairs at known transcriptional boundaries, and non-operon genes from triplet data where genes are transcribed in opposite directions. Click here for file

Additional file 2 *Repressor and Activator site footprint data*. This file contains full details of all the 211 repressor sites, 77 activator sites and 341 dual regulator sites taken footprint data in RegulonDB for E. coli and used in this study for analysing trends in palindromicity. Click here for file
