# Supplementary material for: Predicted transcription factor binding sites as predictors of operons in Escherichia coli and Streptomyces coelicolor
Source: BMC Genomics. 2008 Feb 12;9:79. doi: 10.1186/1471-2164-9-79 (PMC2276206; doi:10.1186/1471-2164-9-79)
Supplement: Additional file 1 — Operon definitions used in this study. This file contains list of operonic and non-operonic genes used in the datasets tested in this study, for Streptomyces coelicolor and E. coli. Data sets are divided into known operons, non-operon gene pairs at known transcriptional boundaries, and non-operon genes from triplet data where genes are transcribed in opposite directions. [file 1471-2164-9-79-S1.doc]

# Additional data file 1

## Operon examples

## S.1.1 *Streptomyces coelicolor* known (positive) operon examples

SCO3671 SCO3670 SCO3669 SCO3668

SCO4761 SCO4762

SCO4648 SCO4649

SCO4652 SCO4653

SCO4654 SCO4655

SCO4659 SCO4660 SCO4661 SCO4662

SCO3229 SCO3228 SCO3227

SCO3138 SCO3137 SCO3136

SCO2619 SCO2618

SCO7281 SCO7280

SCO5877 SCO5878 SCO5879 SCO5880

SCO5887 SCO5886 SCO5883

SCO5888 SCO5889 SCO5890 SCO5891 SCO5892 SCO5893 SCO5894 SCO5895 SCO5896 SCO5897 SCO5898

SCO0185 SCO0186 SCO0187 SCO0188

SCO0191 SCO0190 SCO0189

SCO2231 SCO2230 SCO2229

SCO0713 SCO0712

SCO1439 SCO1438

SCO2054 SCO2053 SCO2052 SCO2051 SCO2050

SCO1580 SCO1579 SCO1578 SCO1577 SCO1576

SCO3214 SCO3213 SCO3212 SCO3211

SCO2387 SCO2388 SCO2389 SCO2390

SCO5354 SCO5355 SCO5356

SCO2611 SCO2610 SCO2609

SCO5512 SCO5513 SCO5514

SCO5032 SCO5031

SCO6683 SCO6684

SCO5443 SCO5442 SCO5441 SCO5440

SCO5624 SCO5625

SCO3356 SCO3357 SCO3358 SCO3359

SCO3890 SCO3889

SCO5216 SCO5217

SCO3887 SCO3888

SCO5320 SCO5319 SCO5318 SCO5317 SCO5316 SCO5315 SCO5314

## S.1.2 *Streptomyces coelicolor* negative (transcriptional boundary) operon examples

SCO3672 SCO3671

SCO3668 SCO3667

SCO4760 SCO4761

SCO4647 SCO4648

SCO4649 SCO4650

SCO4651 SCO4652

SCO4653 SCO4654

SCO4655 SCO4656

SCO4658 SCO4659

SCO2620 SCO2619

SCO2618 SCO2617

SCO5898 SCO5899

SCO0184 SCO0185

SCO0192 SCO0191

SCO2229 SCO2228

SCO0654 SCO0655

SCO0658 SCO0659

SCO0648 SCO0649

SCO0652 SCO0653

SCO6498 SCO6499

SCO6502 SCO6503

SCO6504 SCO6505

SCO0712 SCO0711

SCO1440 SCO1439

SCO1439 SCO1438

SCO2050 SCO2049

SCO3215 SCO3214

SCO3211 SCO3210

SCO0589 SCO0588

SCO0585 SCO0584

SCO1161 SCO1160

SCO1631 SCO1630

SCO1627 SCO1626

SCO2878 SCO2879

SCO2882 SCO2883

SCO5292 SCO5293

SCO5541 SCO5540

SCO6070 SCO6069

SCO6797 SCO6798

SCO6944 SCO6943

SCO6940 SCO6939

SCO7423 SCO7422

SCO7419 SCO7418

SCO7466 SCO7467

SCO0219 SCO0220

SCO4946 SCO4947

SCO6536 SCO6535

SCO3875 SCO3874

SCO3873 SCO3872

SCO2386 SCO2387

SCO5353 SCO5354

SCO5356 SCO5357

SCO2612 SCO2611

SCO2609 SCO2608

SCO5511 SCO5512

SCO5514 SCO5515

SCO6682 SCO6683

SCO5625 SCO5626

SCO3355 SCO3356

SCO3891 SCO3890

SCO5215 SCO5216

SCO5217 SCO5218

SCO3886 SCO3887

SCO5314 SCO5313

SCO5803 SCO5802

## S.1.3 *Streptomyces coelicolor* negative (directional) operon examples

SCO0009 SCO0010 SCO0011

SCO0021 SCO0022 SCO0023

SCO0033 SCO0034 SCO0035

SCO0045 SCO0046 SCO0047

SCO0050 SCO0051 SCO0053

SCO0051 SCO0053 SCO0055

SCO0053 SCO0055 SCO0056

SCO0079 SCO0080 SCO0081

SCO0080 SCO0081 SCO0083

SCO0088 SCO0089 SCO0090

SCO0089 SCO0090 SCO0091

SCO0090 SCO0091 SCO0092

SCO0098 SCO0099 SCO0101

SCO0099 SCO0101 SCO0102

SCO0104 SCO0105 SCO0106

SCO0105 SCO0106 SCO0107

SCO0106 SCO0107 SCO0108

SCO0107 SCO0108 SCO0109

SCO0110 SCO0111 SCO0112

SCO0111 SCO0112 SCO0113

SCO0112 SCO0113 SCO0114

SCO0121 SCO0122 SCO0123

SCO0122 SCO0123 SCO0124

SCO0130 SCO0131 SCO0132

SCO0131 SCO0132 SCO0133

SCO0134 SCO0135 SCO0136

SCO0145 SCO0146 SCO0147

SCO0148 SCO0149 SCO0150

SCO0154 SCO0155 SCO0156

SCO0155 SCO0156 SCO0157

SCO0164 SCO0165 SCO0166

SCO0165 SCO0166 SCO0167

SCO0166 SCO0167 SCO0168

SCO0169 SCO0170 SCO0171

SCO0201 SCO0202 SCO0203

SCO0213 SCO0214 SCO0215

SCO0214 SCO0215 SCO0216

SCO0220 SCO0221 SCO0222

SCO0235 SCO0236 SCO0237

SCO0246 SCO0247 SCO0248

SCO0249 SCO0250 SCO0251

SCO0252 SCO0253 SCO0254

SCO0260 SCO0261 SCO0262

SCO0261 SCO0262 SCO0263

SCO0262 SCO0263 SCO0264

SCO0265 SCO0266 SCO0267

SCO0276 SCO0277 SCO0278

SCO0281 SCO0282 SCO0283

SCO0282 SCO0283 SCO0284

SCO0285 SCO0286 SCO0287

SCO0286 SCO0287 SCO0288

SCO0297 SCO0298 SCO0299

SCO0298 SCO0299 SCO0300

SCO0299 SCO0300 SCO0301

SCO0300 SCO0301 SCO0302

SCO0304 SCO0305 SCO0306

SCO0305 SCO0306 SCO0307

SCO0332 SCO0333 SCO0334

SCO0335 SCO0336 SCO0337

SCO0336 SCO0337 SCO0338

SCO0337 SCO0338 SCO0339

SCO0338 SCO0339 SCO0340

SCO0344 SCO0345 SCO0346

SCO0356 SCO0357 SCO0358

SCO0366 SCO0367 SCO0368

SCO0367 SCO0368 SCO0369

SCO0373 SCO0374 SCO0375

SCO0374 SCO0375 SCO0376

SCO0375 SCO0376 SCO0377

SCO0403 SCO0404 SCO0405

SCO0404 SCO0405 SCO0406

SCO0414 SCO0415 SCO0416

SCO0423 SCO0424 SCO0425

SCO0424 SCO0425 SCO0426

SCO0428 SCO0429 SCO0430

SCO0429 SCO0430 SCO0431

SCO0430 SCO0431 SCO0432

SCO0459 SCO0460 SCO0461

SCO0460 SCO0461 SCO0462

SCO0461 SCO0462 SCO0463

SCO0462 SCO0463 SCO0464

SCO0467 SCO0468 SCO0469

SCO0484 SCO0485 SCO0486

SCO0485 SCO0486 SCO0487

SCO0488 SCO0489 SCO0490

SCO0489 SCO0490 SCO0491

SCO0498 SCO0499 SCO0500

SCO0506 SCO0507 SCO0508

SCO0509 SCO0510 SCO0511

SCO0510 SCO0511 SCO0512

SCO0511 SCO0512 SCO0513

SCO0518 SCO0519 SCO0520

SCO0522 SCO0523 SCO0524

SCO0526 SCO0527 SCO0528

SCO0527 SCO0528 SCO0529

SCO0535 SCO0536 SCO0537

SCO0549 SCO0550 SCO0551

SCO0556 SCO0557 SCO0558

SCO0561 SCO0562 SCO0563

SCO0566 SCO0567 SCO0568

SCO0596 SCO0597 SCO0598

SCO0600 SCO0601 SCO0602

SCO0603 SCO0604 SCO0605

SCO0613 SCO0614 SCO0615

SCO0614 SCO0615 SCO0616

SCO0619 SCO0620 SCO0621

SCO0633 SCO0634 SCO0635

SCO0640 SCO0641 SCO0642

SCO0670 SCO0671 SCO0672

SCO0671 SCO0672 SCO0673

SCO0677 SCO0678 SCO0679

SCO0678 SCO0679 SCO0680

SCO0679 SCO0680 SCO0681

SCO0680 SCO0681 SCO0682

SCO0685 SCO0686 SCO0687

SCO0686 SCO0687 SCO0688

SCO0691 SCO0692 SCO0693

SCO0695 SCO0696 SCO0697

SCO0696 SCO0697 SCO0698

SCO0713 SCO0714 SCO0715

SCO0714 SCO0715 SCO0716

SCO0731 SCO0732 SCO0733

SCO0734 SCO0735 SCO0736

SCO0737 SCO0738 SCO0739

SCO0741 SCO0742 SCO0743

SCO0742 SCO0743 SCO0744

SCO0743 SCO0744 SCO0745

SCO0744 SCO0745 SCO0746

SCO0745 SCO0746 SCO0747

SCO0746 SCO0747 SCO0748

SCO0747 SCO0748 SCO0749

SCO0761 SCO0762 SCO0763

SCO0762 SCO0763 SCO0764

SCO0771 SCO0772 SCO0773

SCO0774 SCO0775 SCO0776

SCO0775 SCO0776 SCO0777

SCO0778 SCO0779 SCO0780

SCO0781 SCO0782 SCO0783

SCO0784 SCO0785 SCO0786

SCO0787 SCO0788 SCO0789

SCO0800 SCO0801 SCO0802

SCO0826 SCO0827 SCO0828

SCO0837 SCO0838 SCO0839

SCO0845 SCO0846 SCO0847

SCO0849 SCO0850 SCO0851

SCO0856 SCO0857 SCO0858

SCO0880 SCO0881 SCO0882

SCO0883 SCO0884 SCO0885

SCO0884 SCO0885 SCO0886

SCO0887 SCO0888 SCO0889

SCO0890 SCO0891 SCO0892

SCO0891 SCO0892 SCO0893

SCO0907 SCO0908 SCO0909

SCO0908 SCO0909 SCO0910

SCO0913 SCO0914 SCO0915

SCO0914 SCO0915 SCO0916

SCO0915 SCO0916 SCO0917

SCO0924 SCO0925 SCO0926

SCO0925 SCO0926 SCO0927

SCO0926 SCO0927 SCO0928

SCO0933 SCO0934 SCO0935

SCO0934 SCO0935 SCO0936

SCO0935 SCO0936 SCO0937

SCO0938 SCO0939 SCO0940

SCO0939 SCO0940 SCO0941

SCO0940 SCO0941 SCO0942

SCO0946 SCO0947 SCO0948

SCO0952 SCO0953 SCO0954

SCO0955 SCO0956 SCO0957

SCO0973 SCO0974 SCO0975

SCO0974 SCO0975 SCO0976

SCO0979 SCO0980 SCO0981

SCO0980 SCO0981 SCO0982

SCO1000 SCO1001 SCO1002

SCO1001 SCO1002 SCO1003

SCO1002 SCO1003 SCO1004

SCO1003 SCO1004 SCO1005

SCO1004 SCO1005 SCO1006

SCO1007 SCO1008 SCO1009

SCO1008 SCO1009 SCO1010

SCO1009 SCO1010 SCO1011

SCO1018 SCO1019 SCO1020

SCO1019 SCO1020 SCO1021

SCO1024 SCO1025 SCO1026

SCO1027 SCO1028 SCO1029

SCO1040 SCO1041 SCO1042

SCO1041 SCO1042 SCO1043

SCO1042 SCO1043 SCO1044

SCO1043 SCO1044 SCO1045

SCO1053 SCO1054 SCO1055

SCO1054 SCO1055 SCO1056

SCO1071 SCO1072 SCO1073

SCO1072 SCO1073 SCO1074

SCO1073 SCO1074 SCO1075

SCO1084 SCO1085 SCO1086

SCO1089 SCO1090 SCO1091

SCO1090 SCO1091 SCO1092

SCO1104 SCO1105 SCO1106

SCO1105 SCO1106 SCO1107

SCO1110 SCO1111 SCO1112

SCO1121 SCO1122 SCO1123

SCO1122 SCO1123 SCO1124

SCO1134 SCO1135 SCO1136

SCO1140 SCO1141 SCO1142

SCO1141 SCO1142 SCO1143

SCO1144 SCO1145 SCO1146

SCO1145 SCO1146 SCO1147

SCO1150 SCO1151 SCO1152

SCO1155 SCO1156 SCO1157

SCO1161 SCO1162 SCO1163

SCO1162 SCO1163 SCO1164

SCO1165 SCO1166 SCO1167

SCO1168 SCO1169 SCO1170

SCO1176 SCO1177 SCO1178

SCO1191 SCO1192 SCO1193

SCO1192 SCO1193 SCO1194

SCO1193 SCO1194 SCO1195

SCO1208 SCO1209 SCO1210

SCO1209 SCO1210 SCO1211

SCO1210 SCO1211 SCO1212

SCO1223 SCO1224 SCO1225

SCO1224 SCO1225 SCO1226

SCO1240 SCO1241 SCO1242

SCO1241 SCO1242 SCO1243

SCO1242 SCO1243 SCO1244

SCO1263 SCO1264 SCO1265

SCO1293 SCO1294 SCO1295

SCO1298 SCO1299 SCO1300

SCO1303 SCO1304 SCO1305

SCO1307 SCO1308 SCO1309

SCO1308 SCO1309 SCO1310

SCO1309 SCO1310 SCO1311

SCO1312 SCO1313 SCO1314

SCO1313 SCO1314 SCO1315

SCO1314 SCO1315 SCO1316

SCO1322 SCO1323 SCO1324

SCO1323 SCO1324 SCO1325

SCO1330 SCO1331 SCO1332

SCO1331 SCO1332 SCO1333

SCO1334 SCO1335 SCO1336

SCO1335 SCO1336 SCO1337

SCO1346 SCO1347 SCO1348

SCO1351 SCO1352 SCO1353

SCO1352 SCO1353 SCO1354

SCO1357 SCO1358 SCO1359

SCO1358 SCO1359 SCO1360

SCO1359 SCO1360 SCO1361

SCO1360 SCO1361 SCO1362

SCO1364 SCO1365 SCO1366

SCO1377 SCO1378 SCO1379

SCO1378 SCO1379 SCO1380

SCO1393 SCO1394 SCO1395

SCO1402 SCO1403 SCO1404

SCO1416 SCO1417 SCO1418

SCO1427 SCO1428 SCO1429

SCO1428 SCO1429 SCO1430

SCO1446 SCO1447 SCO1448

SCO1447 SCO1448 SCO1449

SCO1448 SCO1449 SCO1450

SCO1449 SCO1450 SCO1451

SCO1450 SCO1451 SCO1452

SCO1473 SCO1474 SCO1475

SCO1488 SCO1489 SCO1490

SCO1503 SCO1504 SCO1505

SCO1533 SCO1534 SCO1535

SCO1542 SCO1543 SCO1544

SCO1543 SCO1544 SCO1545

SCO1550 SCO1551 SCO1552

SCO1565 SCO1566 SCO1567

SCO1580 SCO1581 SCO1582

SCO1583 SCO1584 SCO1585

SCO1589 SCO1590 SCO1591

SCO1590 SCO1591 SCO1592

SCO1600 SCO1601 SCO1602

SCO1601 SCO1602 SCO1603

SCO1602 SCO1603 SCO1604

SCO1603 SCO1604 SCO1605

SCO1608 SCO1609 SCO1610

SCO1615 SCO1616 SCO1617

SCO1616 SCO1617 SCO1618

SCO1623 SCO1624 SCO1625

SCO1624 SCO1625 SCO1626

SCO1641 SCO1642 SCO1643

SCO1648 SCO1649 SCO1650

SCO1666 SCO1667 SCO1668

SCO1671 SCO1672 SCO1673

SCO1672 SCO1673 SCO1674

SCO1676 SCO1677 SCO1678

SCO1677 SCO1678 SCO1679

SCO1696 SCO1697 SCO1698

SCO1697 SCO1698 SCO1699

SCO1717 SCO1718 SCO1719

SCO1723 SCO1724 SCO1725

SCO1724 SCO1725 SCO1726

SCO1727 SCO1728 SCO1729

SCO1730 SCO1731 SCO1732

SCO1731 SCO1732 SCO1733

SCO1732 SCO1733 SCO1734

SCO1733 SCO1734 SCO1735

SCO1736 SCO1737 SCO1738

SCO1739 SCO1740 SCO1741

SCO1740 SCO1741 SCO1742

SCO1748 SCO1749 SCO1750

SCO1783 SCO1784 SCO1785

SCO1788 SCO1789 SCO1790

SCO1798 SCO1799 SCO1800

SCO1805 SCO1806 SCO1807

SCO1812 SCO1813 SCO1814

SCO1818 SCO1819 SCO1820

SCO1819 SCO1820 SCO1821

SCO1836 SCO1837 SCO1838

SCO1840 SCO1841 SCO1842

SCO1853 SCO1854 SCO1855

SCO1891 SCO1892 SCO1893

SCO1907 SCO1908 SCO1909

SCO1911 SCO1912 SCO1913

SCO1912 SCO1913 SCO1914

SCO1953 SCO1954 SCO1955

SCO1954 SCO1955 SCO1956

SCO1957 SCO1958 SCO1959

SCO1969 SCO1970 SCO1971

SCO1972 SCO1973 SCO1974

SCO1973 SCO1974 SCO1975

SCO1974 SCO1975 SCO1976

SCO1990 SCO1991 SCO1992

SCO1993 SCO1994 SCO1995

SCO2014 SCO2015 SCO2016

SCO2021 SCO2022 SCO2023

SCO2044 SCO2045 SCO2046

SCO2062 SCO2063 SCO2064

SCO2063 SCO2064 SCO2065

SCO2068 SCO2069 SCO2070

SCO2069 SCO2070 SCO2071

SCO2075 SCO2076 SCO2077

SCO2111 SCO2112 SCO2113

SCO2112 SCO2113 SCO2114

SCO2130 SCO2131 SCO2132

SCO2131 SCO2132 SCO2133

SCO2132 SCO2133 SCO2134

SCO2151 SCO2152 SCO2153

SCO2156 SCO2157 SCO2158

SCO2157 SCO2158 SCO2159

SCO2160 SCO2161 SCO2162

SCO2171 SCO2172 SCO2173

SCO2172 SCO2173 SCO2174

SCO2173 SCO2174 SCO2175

SCO2174 SCO2175 SCO2176

SCO2175 SCO2176 SCO2177

SCO2176 SCO2177 SCO2178

SCO2177 SCO2178 SCO2179

SCO2186 SCO2187 SCO2188

SCO2187 SCO2188 SCO2189

SCO2188 SCO2189 SCO2190

SCO2191 SCO2192 SCO2193

SCO2196 SCO2197 SCO2198

SCO2199 SCO2200 SCO2201

SCO2204 SCO2205 SCO2206

SCO2207 SCO2208 SCO2209

SCO2217 SCO2218 SCO2219

SCO2233 SCO2234 SCO2235

SCO2240 SCO2241 SCO2242

SCO2241 SCO2242 SCO2243

SCO2242 SCO2243 SCO2244

SCO2258 SCO2259 SCO2260

SCO2259 SCO2260 SCO2261

SCO2264 SCO2265 SCO2266

SCO2284 SCO2285 SCO2286

SCO2285 SCO2286 SCO2287

SCO2286 SCO2287 SCO2288

SCO2287 SCO2288 SCO2289

SCO2303 SCO2304 SCO2305

SCO2308 SCO2309 SCO2310

SCO2318 SCO2319 SCO2320

SCO2319 SCO2320 SCO2321

SCO2326 SCO2327 SCO2328

SCO2327 SCO2328 SCO2329

SCO2328 SCO2329 SCO2330

SCO2329 SCO2330 SCO2331

SCO2330 SCO2331 SCO2332

SCO2334 SCO2335 SCO2336

SCO2338 SCO2339 SCO2340

SCO2339 SCO2340 SCO2341

SCO2342 SCO2343 SCO2344

SCO2343 SCO2344 SCO2345

SCO2344 SCO2345 SCO2346

SCO2359 SCO2360 SCO2361

SCO2363 SCO2364 SCO2365

SCO2370 SCO2371 SCO2372

SCO2384 SCO2385 SCO2386

SCO2390 SCO2391 SCO2392

SCO2399 SCO2400 SCO2401

SCO2426 SCO2427 SCO2428

SCO2427 SCO2428 SCO2429

SCO2428 SCO2429 SCO2430

SCO2429 SCO2430 SCO2431

SCO2440 SCO2441 SCO2442

SCO2441 SCO2442 SCO2443

SCO2445 SCO2446 SCO2447

SCO2458 SCO2459 SCO2460

SCO2459 SCO2460 SCO2461

SCO2464 SCO2465 SCO2466

SCO2480 SCO2481 SCO2482

SCO2484 SCO2485 SCO2486

SCO2490 SCO2491 SCO2492

SCO2491 SCO2492 SCO2493

SCO2496 SCO2497 SCO2498

SCO2497 SCO2498 SCO2499

SCO2498 SCO2499 SCO2500

SCO2499 SCO2500 SCO2501

SCO2500 SCO2501 SCO2502

SCO2503 SCO2504 SCO2505

SCO2525 SCO2526 SCO2527

SCO2535 SCO2536 SCO2537

SCO2550 SCO2551 SCO2552

SCO2562 SCO2563 SCO2564

SCO2581 SCO2582 SCO2583

SCO2602 SCO2603 SCO2604

SCO2603 SCO2604 SCO2605

SCO2604 SCO2605 SCO2606

SCO2615 SCO2616 SCO2617

SCO2621 SCO2622 SCO2623

SCO2624 SCO2625 SCO2626

SCO2629 SCO2630 SCO2631

SCO2633 SCO2634 SCO2635

SCO2641 SCO2642 SCO2643

SCO2642 SCO2643 SCO2644

SCO2646 SCO2647 SCO2648

SCO2654 SCO2655 SCO2656

SCO2669 SCO2670 SCO2671

SCO2677 SCO2678 SCO2679

SCO2678 SCO2679 SCO2680

SCO2696 SCO2697 SCO2698

SCO2713 SCO2714 SCO2715

SCO2714 SCO2715 SCO2716

SCO2718 SCO2719 SCO2720

SCO2724 SCO2725 SCO2726

SCO2741 SCO2742 SCO2743

SCO2742 SCO2743 SCO2744

SCO2743 SCO2744 SCO2745

SCO2758 SCO2759 SCO2760

SCO2759 SCO2760 SCO2761

SCO2770 SCO2771 SCO2772

SCO2785 SCO2786 SCO2787

SCO2793 SCO2794 SCO2795

SCO2801 SCO2802 SCO2803

SCO2804 SCO2805 SCO2806

SCO2805 SCO2806 SCO2807

SCO2810 SCO2811 SCO2812

SCO2813 SCO2814 SCO2815

SCO2816 SCO2817 SCO2818

SCO2817 SCO2818 SCO2819

SCO2826 SCO2827 SCO2828

SCO2839 SCO2840 SCO2841

SCO2860 SCO2861 SCO2862

SCO2861 SCO2862 SCO2863

SCO2862 SCO2863 SCO2864

SCO2863 SCO2864 SCO2865

SCO2886 SCO2887 SCO2888

SCO2890 SCO2892 SCO2893

SCO2896 SCO2897 SCO2898

SCO2900 SCO2901 SCO2902

SCO2907 SCO2908 SCO2909

SCO2908 SCO2909 SCO2910

SCO2920 SCO2921 SCO2922

SCO2923 SCO2924 SCO2925

SCO2924 SCO2925 SCO2926

SCO2925 SCO2926 SCO2927

SCO2926 SCO2927 SCO2928

SCO2927 SCO2928 SCO2929

SCO2928 SCO2929 SCO2930

SCO2937 SCO2938 SCO2939

SCO2948 SCO2949 SCO2950

SCO2955 SCO2956 SCO2957

SCO2964 SCO2965 SCO2966

SCO2969 SCO2970 SCO2971

SCO2974 SCO2975 SCO2976

SCO2975 SCO2976 SCO2977

SCO2985 SCO2986 SCO2987

SCO2992 SCO2993 SCO2994

SCO2993 SCO2994 SCO2995

SCO3003 SCO3004 SCO3005

SCO3004 SCO3005 SCO3006

SCO3019 SCO3020 SCO3021

SCO3020 SCO3021 SCO3022

SCO3030 SCO3031 SCO3032

SCO3047 SCO3048 SCO3049

SCO3048 SCO3049 SCO3050

SCO3051 SCO3052 SCO3053

SCO3060 SCO3061 SCO3062

SCO3067 SCO3068 SCO3069

SCO3075 SCO3076 SCO3077

SCO3076 SCO3077 SCO3078

SCO3080 SCO3081 SCO3082

SCO3081 SCO3082 SCO3083

SCO3085 SCO3086 SCO3087

SCO3106 SCO3107 SCO3108

SCO3107 SCO3108 SCO3109

SCO3112 SCO3113 SCO3114

SCO3126 SCO3127 SCO3128

SCO3132 SCO3133 SCO3134

SCO3133 SCO3134 SCO3135

SCO3134 SCO3135 SCO3136

SCO3157 SCO3158 SCO3159

SCO3167 SCO3168 SCO3169

SCO3172 SCO3173 SCO3174

SCO3173 SCO3174 SCO3175

SCO3174 SCO3175 SCO3176

SCO3182 SCO3183 SCO3184

SCO3183 SCO3184 SCO3185

SCO3190 SCO3191 SCO3192

SCO3199 SCO3200 SCO3201

SCO3202 SCO3203 SCO3204

SCO3203 SCO3204 SCO3205

SCO3204 SCO3205 SCO3206

SCO3266 SCO3267 SCO3268

SCO3267 SCO3268 SCO3269

SCO3271 SCO3272 SCO3273

SCO3274 SCO3275 SCO3276

SCO3275 SCO3276 SCO3277

SCO3276 SCO3277 SCO3278

SCO3280 SCO3281 SCO3282

SCO3281 SCO3282 SCO3283

SCO3282 SCO3283 SCO3284

SCO3283 SCO3284 SCO3285

SCO3298 SCO3299 SCO3300

SCO3302 SCO3303 SCO3304

SCO3309 SCO3310 SCO3311

SCO3310 SCO3311 SCO3312

SCO3311 SCO3312 SCO3313

SCO3314 SCO3315 SCO3316

SCO3333 SCO3334 SCO3335

SCO3334 SCO3335 SCO3336

SCO3335 SCO3336 SCO3337

SCO3343 SCO3344 SCO3345

SCO3348 SCO3349 SCO3350

SCO3349 SCO3350 SCO3351

SCO3352 SCO3353 SCO3354

SCO3366 SCO3367 SCO3368

SCO3367 SCO3368 SCO3369

SCO3372 SCO3373 SCO3374

SCO3373 SCO3374 SCO3375

SCO3390 SCO3391 SCO3392

SCO3391 SCO3392 SCO3393

SCO3410 SCO3411 SCO3412

SCO3411 SCO3412 SCO3413

SCO3422 SCO3423 SCO3424

SCO3431 SCO3432 SCO3433

SCO3450 SCO3451 SCO3452

SCO3456 SCO3457 SCO3458

SCO3509 SCO3510 SCO3511

SCO3528 SCO3529 SCO3530

SCO3538 SCO3539 SCO3540

SCO3549 SCO3550 SCO3551

SCO3557 SCO3558 SCO3559

SCO3558 SCO3559 SCO3560

SCO3559 SCO3560 SCO3561

SCO3560 SCO3561 SCO3562

SCO3578 SCO3579 SCO3580

SCO3579 SCO3580 SCO3581

SCO3580 SCO3581 SCO3582

SCO3583 SCO3584 SCO3585

SCO3584 SCO3585 SCO3586

SCO3587 SCO3588 SCO3589

SCO3608 SCO3609 SCO3610

SCO3609 SCO3610 SCO3611

SCO3610 SCO3611 SCO3612

SCO3615 SCO3616 SCO3617

SCO3619 SCO3620 SCO3621

SCO3620 SCO3621 SCO3622

SCO3631 SCO3632 SCO3633

SCO3636 SCO3637 SCO3638

SCO3657 SCO3658 SCO3659

SCO3678 SCO3679 SCO3680

SCO3679 SCO3680 SCO3681

SCO3684 SCO3685 SCO3686

SCO3685 SCO3686 SCO3687

SCO3688 SCO3689 SCO3690

SCO3695 SCO3696 SCO3697

SCO3722 SCO3723 SCO3724

SCO3726 SCO3727 SCO3728

SCO3738 SCO3739 SCO3740

SCO3746 SCO3747 SCO3748

SCO3768 SCO3769 SCO3770

SCO3769 SCO3770 SCO3771

SCO3774 SCO3775 SCO3776

SCO3781 SCO3782 SCO3783

SCO3782 SCO3783 SCO3784

SCO3789 SCO3790 SCO3791

SCO3795 SCO3796 SCO3797

SCO3802 SCO3803 SCO3804

SCO3807 SCO3808 SCO3809

SCO3819 SCO3820 SCO3821

SCO3820 SCO3821 SCO3822

SCO3831 SCO3832 SCO3833

SCO3834 SCO3835 SCO3836

SCO3853 SCO3854 SCO3855

SCO3856 SCO3857 SCO3858

SCO3857 SCO3858 SCO3859

SCO3869 SCO3870 SCO3871

SCO3870 SCO3871 SCO3872

SCO3895 SCO3896 SCO3897

SCO3909 SCO3910 SCO3911

SCO3926 SCO3927 SCO3928

SCO3927 SCO3928 SCO3929

SCO3928 SCO3929 SCO3930

SCO3931 SCO3932 SCO3933

SCO3936 SCO3937 SCO3938

SCO3941 SCO3942 SCO3943

SCO3942 SCO3943 SCO3944

SCO3976 SCO3977 SCO3978

SCO3977 SCO3978 SCO3979

SCO3978 SCO3979 SCO3980

SCO3997 SCO3998 SCO3999

SCO3998 SCO3999 SCO4000

SCO4001 SCO4002 SCO4003

SCO4006 SCO4007 SCO4008

SCO4007 SCO4008 SCO4009

SCO4010 SCO4011 SCO4012

SCO4011 SCO4012 SCO4013

SCO4012 SCO4013 SCO4014

SCO4013 SCO4014 SCO4015

SCO4032 SCO4033 SCO4034

SCO4043 SCO4044 SCO4045

SCO4054 SCO4055 SCO4056

SCO4055 SCO4056 SCO4057

SCO4056 SCO4057 SCO4058

SCO4060 SCO4061 SCO4062

SCO4061 SCO4062 SCO4063

SCO4079 SCO4080 SCO4081

SCO4097 SCO4098 SCO4099

SCO4111 SCO4112 SCO4113

SCO4120 SCO4121 SCO4122

SCO4121 SCO4122 SCO4123

SCO4124 SCO4125 SCO4126

SCO4133 SCO4134 SCO4135

SCO4134 SCO4135 SCO4136

SCO4135 SCO4136 SCO4137

SCO4150 SCO4151 SCO4152

SCO4151 SCO4152 SCO4153

SCO4152 SCO4153 SCO4154

SCO4153 SCO4154 SCO4155

SCO4166 SCO4167 SCO4168

SCO4186 SCO4187 SCO4188

SCO4187 SCO4188 SCO4189

SCO4188 SCO4189 SCO4190

SCO4195 SCO4196 SCO4197

SCO4205 SCO4206 SCO4207

SCO4206 SCO4207 SCO4208

SCO4207 SCO4208 SCO4209

SCO4208 SCO4209 SCO4210

SCO4213 SCO4214 SCO4215

SCO4214 SCO4215 SCO4216

SCO4221 SCO4222 SCO4223

SCO4230 SCO4231 SCO4232

SCO4267 SCO4268 SCO4269

SCO4276 SCO4277 SCO4278

SCO4277 SCO4278 SCO4279

SCO4289 SCO4290 SCO4291

SCO4290 SCO4291 SCO4292

SCO4291 SCO4292 SCO4293

SCO4301 SCO4302 SCO4303

SCO4302 SCO4303 SCO4304

SCO4303 SCO4304 SCO4305

SCO4315 SCO4316 SCO4317

SCO4316 SCO4317 SCO4318

SCO4323 SCO4324 SCO4325

SCO4324 SCO4325 SCO4326

SCO4333 SCO4334 SCO4335

SCO4334 SCO4335 SCO4336

SCO4338 SCO4339 SCO4340

SCO4344 SCO4345 SCO4346

SCO4354 SCO4355 SCO4356

SCO4355 SCO4356 SCO4357

SCO4356 SCO4357 SCO4358

SCO4357 SCO4358 SCO4359

SCO4365 SCO4366 SCO4367

SCO4366 SCO4367 SCO4368

SCO4367 SCO4368 SCO4369

SCO4386 SCO4387 SCO4388

SCO4390 SCO4391 SCO4392

SCO4391 SCO4392 SCO4393

SCO4392 SCO4393 SCO4394

SCO4400 SCO4401 SCO4402

SCO4406 SCO4407 SCO4408

SCO4412 SCO4413 SCO4414

SCO4415 SCO4416 SCO4417

SCO4416 SCO4417 SCO4418

SCO4419 SCO4420 SCO4421

SCO4420 SCO4421 SCO4422

SCO4430 SCO4431 SCO4432

SCO4431 SCO4432 SCO4433

SCO4432 SCO4433 SCO4434

SCO4459 SCO4460 SCO4461

SCO4460 SCO4461 SCO4462

SCO4463 SCO4464 SCO4465

SCO4464 SCO4465 SCO4466

SCO4477 SCO4478 SCO4479

SCO4480 SCO4481 SCO4482

SCO4481 SCO4482 SCO4483

SCO4484 SCO4485 SCO4486

SCO4488 SCO4489 SCO4490

SCO4507 SCO4508 SCO4509

SCO4508 SCO4509 SCO4510

SCO4518 SCO4519 SCO4520

SCO4551 SCO4552 SCO4553

SCO4558 SCO4559 SCO4560

SCO4575 SCO4576 SCO4577

SCO4576 SCO4577 SCO4578

SCO4580 SCO4581 SCO4582

SCO4581 SCO4582 SCO4583

SCO4586 SCO4587 SCO4588

SCO4587 SCO4588 SCO4589

SCO4590 SCO4591 SCO4592

SCO4611 SCO4612 SCO4613

SCO4612 SCO4613 SCO4614

SCO4622 SCO4623 SCO4624

SCO4631 SCO4632 SCO4633

SCO4637 SCO4638 SCO4639

SCO4656 SCO4657 SCO4658

SCO4673 SCO4674 SCO4675

SCO4679 SCO4680 SCO4681

SCO4731 SCO4732 SCO4733

SCO4742 SCO4743 SCO4744

SCO4743 SCO4744 SCO4745

SCO4748 SCO4749 SCO4750

SCO4757 SCO4758 SCO4759

SCO4765 SCO4766 SCO4767

SCO4766 SCO4767 SCO4768

SCO4772 SCO4773 SCO4774

SCO4781 SCO4782 SCO4783

SCO4782 SCO4783 SCO4784

SCO4783 SCO4784 SCO4785

SCO4784 SCO4785 SCO4786

SCO4785 SCO4786 SCO4787

SCO4789 SCO4790 SCO4791

SCO4792 SCO4793 SCO4794

SCO4793 SCO4794 SCO4795

SCO4796 SCO4797 SCO4798

SCO4799 SCO4800 SCO4801

SCO4809 SCO4810 SCO4811

SCO4810 SCO4811 SCO4812

SCO4811 SCO4812 SCO4813

SCO4837 SCO4838 SCO4839

SCO4846 SCO4847 SCO4848

SCO4867 SCO4868 SCO4869

SCO4868 SCO4869 SCO4870

SCO4869 SCO4870 SCO4871

SCO4870 SCO4871 SCO4872

SCO4890 SCO4891 SCO4892

SCO4891 SCO4892 SCO4893

SCO4896 SCO4897 SCO4898

SCO4897 SCO4898 SCO4899

SCO4898 SCO4899 SCO4900

SCO4899 SCO4900 SCO4901

SCO4907 SCO4908 SCO4909

SCO4908 SCO4909 SCO4910

SCO4914 SCO4915 SCO4916

SCO4920 SCO4921 SCO4922

SCO4921 SCO4922 SCO4923

SCO4928 SCO4929 SCO4930

SCO4936 SCO4937 SCO4938

SCO4937 SCO4938 SCO4939

SCO4938 SCO4939 SCO4940

SCO4939 SCO4940 SCO4941

SCO4942 SCO4943 SCO4944

SCO4943 SCO4944 SCO4945

SCO4950 SCO4951 SCO4952

SCO4955 SCO4956 SCO4957

SCO4956 SCO4957 SCO4958

SCO4957 SCO4958 SCO4959

SCO4978 SCO4979 SCO4980

SCO4981 SCO4982 SCO4983

SCO4984 SCO4985 SCO4986

SCO5015 SCO5016 SCO5017

SCO5018 SCO5019 SCO5020

SCO5026 SCO5027 SCO5028

SCO5032 SCO5033 SCO5034

SCO5038 SCO5039 SCO5040

SCO5046 SCO5047 SCO5048

SCO5047 SCO5048 SCO5049

SCO5048 SCO5049 SCO5050

SCO5059 SCO5060 SCO5061

SCO5060 SCO5061 SCO5062

SCO5063 SCO5064 SCO5065

SCO5064 SCO5065 SCO5066

SCO5067 SCO5068 SCO5069

SCO5068 SCO5069 SCO5070

SCO5081 SCO5082 SCO5083

SCO5085 SCO5086 SCO5087

SCO5094 SCO5095 SCO5096

SCO5095 SCO5096 SCO5097

SCO5121 SCO5122 SCO5123

SCO5122 SCO5123 SCO5124

SCO5140 SCO5141 SCO5142

SCO5143 SCO5144 SCO5145

SCO5144 SCO5145 SCO5146

SCO5145 SCO5146 SCO5147

SCO5154 SCO5155 SCO5156

SCO5157 SCO5158 SCO5159

SCO5158 SCO5159 SCO5160

SCO5162 SCO5163 SCO5164

SCO5163 SCO5164 SCO5165

SCO5166 SCO5167 SCO5168

SCO5186 SCO5187 SCO5188

SCO5202 SCO5203 SCO5204

SCO5208 SCO5209 SCO5210

SCO5241 SCO5242 SCO5243

SCO5245 SCO5246 SCO5247

SCO5253 SCO5254 SCO5255

SCO5254 SCO5255 SCO5256

SCO5255 SCO5256 SCO5257

SCO5256 SCO5257 SCO5258

SCO5265 SCO5266 SCO5267

SCO5266 SCO5267 SCO5268

SCO5284 SCO5285 SCO5286

SCO5285 SCO5286 SCO5287

SCO5286 SCO5287 SCO5288

SCO5287 SCO5288 SCO5289

SCO5296 SCO5297 SCO5299

SCO5306 SCO5307 SCO5308

SCO5307 SCO5308 SCO5309

SCO5308 SCO5309 SCO5310

SCO5322 SCO5323 SCO5324

SCO5325 SCO5326 SCO5327

SCO5326 SCO5327 SCO5328

SCO5327 SCO5328 SCO5329

SCO5330 SCO5331 SCO5332

SCO5331 SCO5332 SCO5333

SCO5334 SCO5335 SCO5336

SCO5387 SCO5388 SCO5389

SCO5388 SCO5389 SCO5390

SCO5389 SCO5390 SCO5391

SCO5390 SCO5391 SCO5392

SCO5401 SCO5402 SCO5403

SCO5408 SCO5409 SCO5410

SCO5415 SCO5416 SCO5417

SCO5416 SCO5417 SCO5418

SCO5417 SCO5418 SCO5419

SCO5425 SCO5426 SCO5427

SCO5443 SCO5444 SCO5445

SCO5455 SCO5456 SCO5457

SCO5461 SCO5462 SCO5463

SCO5465 SCO5466 SCO5467

SCO5466 SCO5467 SCO5468

SCO5482 SCO5483 SCO5484

SCO5486 SCO5487 SCO5488

SCO5487 SCO5488 SCO5489

SCO5515 SCO5516 SCO5517

SCO5516 SCO5517 SCO5518

SCO5517 SCO5518 SCO5519

SCO5523 SCO5524 SCO5525

SCO5530 SCO5531 SCO5532

SCO5531 SCO5532 SCO5533

SCO5532 SCO5533 SCO5534

SCO5549 SCO5550 SCO5551

SCO5550 SCO5551 SCO5552

SCO5551 SCO5552 SCO5553

SCO5560 SCO5561 SCO5562

SCO5563 SCO5564 SCO5565

SCO5578 SCO5579 SCO5580

SCO5579 SCO5580 SCO5581

SCO5580 SCO5581 SCO5582

SCO5605 SCO5606 SCO5607

SCO5622 SCO5623 SCO5624

SCO5636 SCO5637 SCO5638

SCO5664 SCO5665 SCO5666

SCO5673 SCO5674 SCO5675

SCO5674 SCO5675 SCO5676

SCO5675 SCO5676 SCO5677

SCO5684 SCO5685 SCO5686

SCO5691 SCO5692 SCO5693

SCO5717 SCO5718 SCO5719

SCO5722 SCO5723 SCO5724

SCO5746 SCO5747 SCO5748

SCO5764 SCO5765 SCO5766

SCO5767 SCO5768 SCO5769

SCO5779 SCO5780 SCO5781

SCO5780 SCO5781 SCO5782

SCO5785 SCO5786 SCO5787

SCO5802 SCO5803 SCO5804

SCO5805 SCO5806 SCO5807

SCO5809 SCO5810 SCO5811

SCO5813 SCO5814 SCO5815

SCO5831 SCO5832 SCO5833

SCO5834 SCO5835 SCO5836

SCO5835 SCO5836 SCO5837

SCO5840 SCO5841 SCO5842

SCO5845 SCO5846 SCO5847

SCO5852 SCO5853 SCO5854

SCO5853 SCO5854 SCO5855

SCO5854 SCO5855 SCO5856

SCO5855 SCO5856 SCO5857

SCO5865 SCO5866 SCO5867

SCO5905 SCO5906 SCO5907

SCO5906 SCO5907 SCO5908

SCO5910 SCO5911 SCO5912

SCO5913 SCO5914 SCO5915

SCO5929 SCO5930 SCO5931

SCO5930 SCO5931 SCO5932

SCO5931 SCO5932 SCO5933

SCO5932 SCO5933 SCO5934

SCO5949 SCO5950 SCO5951

SCO5954 SCO5955 SCO5956

SCO5964 SCO5965 SCO5966

SCO5965 SCO5966 SCO5967

SCO5973 SCO5974 SCO5975

SCO5977 SCO5978 SCO5979

SCO5986 SCO5987 SCO5988

SCO5987 SCO5988 SCO5989

SCO5997 SCO5998 SCO5999

SCO6011 SCO6012 SCO6013

SCO6014 SCO6015 SCO6016

SCO6019 SCO6020 SCO6021

SCO6030 SCO6031 SCO6032

SCO6031 SCO6032 SCO6033

SCO6074 SCO6075 SCO6076

SCO6077 SCO6078 SCO6079

SCO6084 SCO6085 SCO6086

SCO6090 SCO6091 SCO6092

SCO6091 SCO6092 SCO6093

SCO6120 SCO6121 SCO6122

SCO6121 SCO6122 SCO6123

SCO6122 SCO6123 SCO6124

SCO6125 SCO6126 SCO6127

SCO6126 SCO6127 SCO6128

SCO6129 SCO6130 SCO6131

SCO6140 SCO6141 SCO6142

SCO6143 SCO6144 SCO6145

SCO6146 SCO6147 SCO6148

SCO6158 SCO6159 SCO6160

SCO6190 SCO6191 SCO6192

SCO6191 SCO6192 SCO6193

SCO6196 SCO6197 SCO6198

SCO6199 SCO6200 SCO6201

SCO6200 SCO6201 SCO6202

SCO6221 SCO6222 SCO6223

SCO6222 SCO6223 SCO6224

SCO6244 SCO6245 SCO6246

SCO6245 SCO6246 SCO6247

SCO6248 SCO6249 SCO6250

SCO6251 SCO6252 SCO6253

SCO6254 SCO6255 SCO6256

SCO6264 SCO6265 SCO6266

SCO6271 SCO6272 SCO6273

SCO6281 SCO6282 SCO6283

SCO6285 SCO6286 SCO6287

SCO6293 SCO6294 SCO6295

SCO6294 SCO6295 SCO6296

SCO6301 SCO6302 SCO6303

SCO6316 SCO6317 SCO6318

SCO6317 SCO6318 SCO6319

SCO6318 SCO6319 SCO6320

SCO6319 SCO6320 SCO6321

SCO6322 SCO6323 SCO6324

SCO6323 SCO6324 SCO6325

SCO6335 SCO6336 SCO6337

SCO6343 SCO6344 SCO6345

SCO6347 SCO6348 SCO6349

SCO6348 SCO6349 SCO6350

SCO6349 SCO6350 SCO6351

SCO6350 SCO6351 SCO6352

SCO6368 SCO6369 SCO6370

SCO6369 SCO6370 SCO6371

SCO6375 SCO6376 SCO6377

SCO6381 SCO6382 SCO6383

SCO6391 SCO6392 SCO6393

SCO6396 SCO6399 SCO6400

SCO6399 SCO6400 SCO6401

SCO6400 SCO6401 SCO6402

SCO6401 SCO6402 SCO6403

SCO6402 SCO6403 SCO6404

SCO6405 SCO6406 SCO6407

SCO6406 SCO6407 SCO6408

SCO6427 SCO6428 SCO6429

SCO6438 SCO6439 SCO6440

SCO6446 SCO6447 SCO6448

SCO6455 SCO6456 SCO6457

SCO6458 SCO6459 SCO6460

SCO6465 SCO6466 SCO6467

SCO6475 SCO6476 SCO6477

SCO6490 SCO6491 SCO6492

SCO6491 SCO6492 SCO6493

SCO6492 SCO6493 SCO6494

SCO6493 SCO6494 SCO6495

SCO6494 SCO6495 SCO6496

SCO6511 SCO6512 SCO6513

SCO6512 SCO6513 SCO6514

SCO6513 SCO6514 SCO6515

SCO6537 SCO6538 SCO6539

SCO6538 SCO6539 SCO6540

SCO6542 SCO6543 SCO6544

SCO6543 SCO6544 SCO6545

SCO6553 SCO6554 SCO6555

SCO6554 SCO6555 SCO6556

SCO6555 SCO6556 SCO6557

SCO6587 SCO6588 SCO6589

SCO6588 SCO6589 SCO6590

SCO6605 SCO6606 SCO6607

SCO6623 SCO6624 SCO6625

SCO6627 SCO6628 SCO6629

SCO6646 SCO6647 SCO6648

SCO6653 SCO6654 SCO6655

SCO6654 SCO6655 SCO6656

SCO6663 SCO6664 SCO6665

SCO6669 SCO6670 SCO6671

SCO6670 SCO6671 SCO6672

SCO6675 SCO6676 SCO6677

SCO6684 SCO6685 SCO6686

SCO6689 SCO6690 SCO6691

SCO6690 SCO6691 SCO6692

SCO6693 SCO6694 SCO6695

SCO6694 SCO6695 SCO6696

SCO6695 SCO6696 SCO6697

SCO6703 SCO6704 SCO6705

SCO6704 SCO6705 SCO6706

SCO6705 SCO6706 SCO6707

SCO6706 SCO6707 SCO6708

SCO6713 SCO6714 SCO6715

SCO6720 SCO6721 SCO6722

SCO6721 SCO6722 SCO6723

SCO6724 SCO6725 SCO6726

SCO6725 SCO6726 SCO6727

SCO6726 SCO6727 SCO6728

SCO6727 SCO6728 SCO6729

SCO6728 SCO6729 SCO6730

SCO6732 SCO6733 SCO6734

SCO6735 SCO6736 SCO6737

SCO6739 SCO6740 SCO6741

SCO6742 SCO6743 SCO6744

SCO6743 SCO6744 SCO6745

SCO6744 SCO6745 SCO6746

SCO6747 SCO6748 SCO6749

SCO6748 SCO6749 SCO6750

SCO6771 SCO6772 SCO6773

SCO6774 SCO6775 SCO6776

SCO6775 SCO6776 SCO6777

SCO6779 SCO6780 SCO6781

SCO6790 SCO6791 SCO6792

SCO6791 SCO6792 SCO6793

SCO6792 SCO6793 SCO6794

SCO6827 SCO6828 SCO6829

SCO6828 SCO6829 SCO6830

SCO6840 SCO6841 SCO6842

SCO6852 SCO6853 SCO6854

SCO6905 SCO6906 SCO6907

SCO6906 SCO6907 SCO6908

SCO6907 SCO6908 SCO6909

SCO6908 SCO6909 SCO6910

SCO6909 SCO6910 SCO6911

SCO6925 SCO6926 SCO6927

SCO6932 SCO6933 SCO6934

SCO6948 SCO6949 SCO6950

SCO6990 SCO6991 SCO6992

SCO6991 SCO6992 SCO6993

SCO6992 SCO6993 SCO6994

SCO6993 SCO6994 SCO6995

SCO7006 SCO7007 SCO7008

SCO7007 SCO7008 SCO7009

SCO7017 SCO7018 SCO7019

SCO7018 SCO7019 SCO7020

SCO7024 SCO7025 SCO7026

SCO7025 SCO7026 SCO7027

SCO7026 SCO7027 SCO7028

SCO7040 SCO7041 SCO7042

SCO7041 SCO7042 SCO7043

SCO7047 SCO7048 SCO7049

SCO7048 SCO7049 SCO7050

SCO7061 SCO7062 SCO7063

SCO7062 SCO7063 SCO7064

SCO7069 SCO7070 SCO7071

SCO7078 SCO7079 SCO7080

SCO7095 SCO7096 SCO7097

SCO7103 SCO7104 SCO7105

SCO7104 SCO7105 SCO7106

SCO7105 SCO7106 SCO7107

SCO7109 SCO7110 SCO7111

SCO7112 SCO7113 SCO7114

SCO7125 SCO7126 SCO7127

SCO7134 SCO7135 SCO7136

SCO7135 SCO7136 SCO7137

SCO7136 SCO7137 SCO7138

SCO7140 SCO7141 SCO7142

SCO7149 SCO7150 SCO7151

SCO7153 SCO7154 SCO7155

SCO7154 SCO7155 SCO7156

SCO7167 SCO7168 SCO7169

SCO7168 SCO7169 SCO7170

SCO7169 SCO7170 SCO7171

SCO7170 SCO7171 SCO7172

SCO7173 SCO7174 SCO7175

SCO7174 SCO7175 SCO7176

SCO7175 SCO7176 SCO7177

SCO7189 SCO7190 SCO7191

SCO7190 SCO7191 SCO7192

SCO7203 SCO7204 SCO7205

SCO7209 SCO7210 SCO7211

SCO7212 SCO7213 SCO7214

SCO7222 SCO7223 SCO7224

SCO7223 SCO7224 SCO7225

SCO7242 SCO7243 SCO7244

SCO7251 SCO7252 SCO7253

SCO7256 SCO7257 SCO7258

SCO7257 SCO7258 SCO7259

SCO7258 SCO7259 SCO7260

SCO7270 SCO7271 SCO7272

SCO7271 SCO7272 SCO7273

SCO7272 SCO7273 SCO7274

SCO7276 SCO7277 SCO7278

SCO7277 SCO7278 SCO7279

SCO7278 SCO7279 SCO7280

SCO7286 SCO7287 SCO7288

SCO7287 SCO7288 SCO7289

SCO7298 SCO7299 SCO7300

SCO7329 SCO7330 SCO7331

SCO7338 SCO7339 SCO7340

SCO7339 SCO7340 SCO7341

SCO7340 SCO7341 SCO7342

SCO7341 SCO7342 SCO7343

SCO7346 SCO7347 SCO7348

SCO7356 SCO7357 SCO7358

SCO7360 SCO7361 SCO7362

SCO7366 SCO7367 SCO7368

SCO7367 SCO7368 SCO7369

SCO7368 SCO7369 SCO7370

SCO7369 SCO7370 SCO7371

SCO7374 SCO7375 SCO7376

SCO7378 SCO7379 SCO7380

SCO7379 SCO7380 SCO7381

SCO7380 SCO7381 SCO7382

SCO7381 SCO7382 SCO7383

SCO7385 SCO7386 SCO7387

SCO7389 SCO7390 SCO7391

SCO7392 SCO7393 SCO7394

SCO7393 SCO7394 SCO7395

SCO7402 SCO7403 SCO7404

SCO7413 SCO7414 SCO7415

SCO7414 SCO7415 SCO7416

SCO7425 SCO7426 SCO7427

SCO7428 SCO7429 SCO7430

SCO7429 SCO7430 SCO7431

SCO7439 SCO7440 SCO7441

SCO7443 SCO7444 SCO7445

SCO7446 SCO7447 SCO7448

SCO7457 SCO7458 SCO7459

SCO7458 SCO7459 SCO7460

SCO7459 SCO7460 SCO7461

SCO7475 SCO7476 SCO7477

SCO7476 SCO7477 SCO7478

SCO7479 SCO7480 SCO7481

SCO7480 SCO7481 SCO7482

SCO7493 SCO7494 SCO7495

SCO7494 SCO7495 SCO7496

SCO7495 SCO7496 SCO7497

SCO7496 SCO7497 SCO7498

SCO7500 SCO7501 SCO7502

SCO7501 SCO7502 SCO7503

SCO7526 SCO7527 SCO7528

SCO7536 SCO7537 SCO7538

SCO7537 SCO7538 SCO7539

SCO7552 SCO7553 SCO7554

SCO7553 SCO7554 SCO7555

SCO7567 SCO7568 SCO7569

SCO7572 SCO7573 SCO7574

SCO7573 SCO7574 SCO7575

SCO7576 SCO7577 SCO7578

SCO7577 SCO7578 SCO7579

SCO7582 SCO7583 SCO7584

SCO7601 SCO7602 SCO7603

SCO7602 SCO7603 SCO7604

SCO7603 SCO7604 SCO7605

SCO7604 SCO7605 SCO7606

SCO7605 SCO7606 SCO7607

SCO7606 SCO7607 SCO7608

SCO7607 SCO7608 SCO7609

SCO7608 SCO7609 SCO7610

SCO7624 SCO7625 SCO7626

SCO7625 SCO7626 SCO7627

SCO7630 SCO7631 SCO7632

SCO7631 SCO7632 SCO7633

SCO7632 SCO7633 SCO7634

SCO7633 SCO7634 SCO7635

SCO7634 SCO7635 SCO7636

SCO7635 SCO7636 SCO7637

SCO7638 SCO7639 SCO7640

SCO7639 SCO7640 SCO7641

SCO7643 SCO7644 SCO7645

SCO7649 SCO7650 SCO7651

SCO7650 SCO7651 SCO7652

SCO7651 SCO7652 SCO7653

SCO7652 SCO7653 SCO7654

SCO7658 SCO7659 SCO7660

SCO7680 SCO7681 SCO7682

SCO7692 SCO7693 SCO7694

SCO7693 SCO7694 SCO7695

SCO7694 SCO7695 SCO7696

SCO7697 SCO7698 SCO7699

SCO7705 SCO7706 SCO7707

SCO7706 SCO7707 SCO7708

SCO7707 SCO7708 SCO7709

SCO7726 SCO7727 SCO7728

SCO7735 SCO7736 SCO7737

SCO7736 SCO7737 SCO7738

SCO7741 SCO7742 SCO7743

SCO7756 SCO7757 SCO7759

SCO7757 SCO7759 SCO7760

SCO7759 SCO7760 SCO7761

SCO7766 SCO7767 SCO7768

SCO7776 SCO7777 SCO7778

SCO7777 SCO7778 SCO7779

SCO7778 SCO7779 SCO7780

SCO7809 SCO7810 SCO7811

SCO7812 SCO7813 SCO7814

SCO7813 SCO7814 SCO7815

SCO7816 SCO7817 SCO7818

SCO7817 SCO7818 SCO7819

SCO7818 SCO7819 SCO7820

SCO7824 SCO7825 SCO7826

## S.1.4 *Escherichia coli* known (positive) operon examples

panB panC

creA creB creC creD

proB proA

tauA tauB tauC tauD

metB metL

secE nusG

acrA acrB

panF prmA

glyQ glyS

metZ metW metV

malK lamB malM

moaA moaB moaC moaD moaE

aroL yaiA aroM

narX narL

valU valX valY lysV

yjeF yjeE amiB mutL miaA hfq hflX hflK hflC

ascF ascB

wza wzb b206 b200 wcaA wcaB

pyrB pyrI

speA speB

lctP lctR lctD

celA celB celC celD celF ydjC

lacZ lacY lacA

ubiC ubiA

gcvT gcvH gcvP

fimB fimE fimA fimI fimC fimD fimF fimG fimH

entC entE entB entA ybdB

deoC deoA deoB deoD

kdtA kdtB

dadA dadX

glgC glgA glgP

alaW alaX

ylcB ylcC ylcD ybdE

yhaU yhaF yhaE yhaD rnpB

phoB phoR

fepD fepG fepC

pqiA pqiB

guaB guaA

trpL trpE trpD trpC trpB trpA

speE speD

nagB nagA nagC nagD

metY yhbC nusA infB rbfA truB rpsO pnp

glnA glnL glnG

uxuA uxuB

mobA mobB

nrdA nrdB

fes entF fepE

fhuA fhuC fhuD fhuB

dcuB fumB

focA pflB

ivbL ilvB ilvN

betI betB betA

cysJ cysI cysH

nikA nikB nikC nikD nikE nikR

motA motB cheA cheW

dnaA dnaN recF

flgB flgC flgD flgE flgF flgG flgH flgI flgJ flgK

thrS infC rpmI rplT

araB araA araD

ycdG ycdH ycdI ycdJ ycdK ycdL ycdM

fliD fliS fliT

fecA fecB fecC fecD fecE

fliA fliZ fliY

pspA pspB pspC pspD pspE

lgt thyA

manX manY manZ

leuQ leuP leuV

rrsA ileT alaT rrlA rrfA

tehA tehB

mtlA mtlD mtlR

rrsC gltU rrlC rrfC

atoD atoA atoE

ylcA ybcZ

rbsD rbsA rbsC rbsB rbsK

pdhR aceE aceF lpdA

treB treC

pheL pheA

hipB hipA

arsR arsB arsC

surE pcm

rrnD ileU alaU rrlD rrfD thrV rrfF

g30K rpmF

fecI fecR

ppk ppx

idnD idnO idnT idnR

tnaL tnaA tnaB

gabD gabT gabP gabC

rpmB rpmG

menF menD yfbB menB menC menE

aceB aceA aceK

lepA lepB

ttdA ttdB

nuoA nuoB nuoC nuoE nuoF nuoG nuoH nuoI nuoJ nuoK nuoL nuoM nuoN

cadB cadA

nrdH nrdI nrdE nrdF

otsB otsA

fepA entD

cydD cydC

chpS chpB

ecpD htrE

mglB mglA mglC

yhdW yhdX yhdY yhdZ

galE galT galK galM

ftsY ftsE ftsX

fldA fur

cyoA cyoB cyoC cyoD cyoE

frdA frdB frdC frdD

napF napD napA napG napH napB napC ccmA ccmB ccmC ccmD ccmE ccmF dsbE ccmH

cysD cysN cysC

yiaK yiaL yiaM yiaN yiaO yiaP yiaQ yiaR yiaS

torY torZ

tdcA tdcB tdcC tdcD tdcE tdcF tdcG

ribF ileS lspA slpA lytB

paaX paaY

oppA oppB oppC oppD oppF

pth ychF

nirB nirD nirC cysG

malP malQ

atpI atpB atpE atpF atpH atpA atpG atpD atpC

clpP clpX

rfaQ rfaG rfaP rfaS rfaB rfaI rfaJ rfaY rfaZ rfaK

glpE glpG glpR

prfB lysS

glyV glyX glyY

kdpD kdpE

sufA sufB sufC sufD sufS sufE

dhaK dhaL dhaM

udk dcd

upp uraA

ptsH ptsI crr

nfrB nfrA

lexA dinF

rrsB gltT rrlB rrfB

rhaB rhaA rhaD

umuD umuC

leuL leuA leuB leuC leuD

minE minD minC

rnc era recO pdxJ acpS

bioB bioF bioC bioD

tolB pal ybgF

rpsU dnaG rpoD

basR basS

rpsB tsf

rplN rplX rplE rpsN rpsH rplF rplR rpsE rpmD rplO prlA rpmJ

amiA hemF

rpsJ rplC rplD rplW rplB rpsS rplV rpsC rplP rpmC rpsQ

malX malY

malE malF malG

glcD glcE glcF glcG glcB glcA

moeA moeB

selA selB

ydcS ydcT ydcU ydcV

mreB mreC mreD yhdE cafA

hycA hycB hycC hycD hycE hycF hycG hycH

cysP cysU cysW cysA cysM

nrfA nrfB nrfC nrfD nrfE nrfF nrfG

ilvI ilvH

glnK amtB

rpoE rseA rseB rseC

hemC hemD hemX hemY

caiT caiA caiB caiC caiD caiE

aroK aroB damX dam rpe gph trpS

astC astA astD astB astE

ada alkB

smtA mukF mukE mukB

thrL thrA thrB thrC

thiC thiE thiF thiG thiH

phoP phoQ

glpT glpQ

pcnB folK

dapA nlpB

dppA dppB dppC dppD dppF

nlpD rpoS

ampD ampE

pstS pstC pstA pstB phoU

modA modB modC

iscR iscS iscU iscA

dmsA dmsB dmsC

ftsJ hflB

ydjA selD topB

yihE dsbA

dnaT dnaC

fixA fixB fixC fixX

fruB fruK fruA

melA melB

gltI gltJ gltK gltL

gntK gntU

srlA srlE srlB srlD gutM srlR gutQ

hscB hscA fdx

rph pyrE

edd eda

livK livH livM livG livF

serC aroA

purH purD

b121 b123 ychA kdsA

hyaA hyaB hyaC hyaD hyaE hyaF

potA potB potC potD

rnt lhr

lysT valT lysW

xylA xylB

epd pgk

pheS pheT himA

rfaD rfaF rfaC rfaL

rpmH rnpA

rhaS rhaR

ddpX ddpA ddpB ddpC ddpD ddpE

cydA cydB

ybgA phrB

yeaG yeaH

ansA ydjB

fliF fliG fliH fliI fliJ fliK

rplJ rplL rpoB rpoC

cmk rpsA

dsdX dsdA

glpA glpB glpC

ebgA ebgC

gatY gatZ gatA gatB gatC gatD gatR gat_2

xylF xylG xylH xylR

argX hisR leuT proM

kdpA kdpB kdpC

rpsM rpsK rpsD rpoA rplQ

exbB exbD

phoA psiF

feoA feoB

rrsG gltW rrlG rrfG

carA carB

fadB fadA

hyb0 hybA hybB hybC hybD hybE hybF hybG

fdnG fdnH fdnI

marR marA marB

rplM rpsI

glmU glmS

phnC phnD phnE f73 phnF phnG phnH phnI phnJ phnK phnL phnM phnN phnO phnP

purE purK

dnaK dnaJ

hdeA hdeB

gadB gadC

thrU tyrU glyT thrT

hslV hslU

cynT cynS cynX

torC torA torD

sdaC sdaB

fucA fucO

ybjC nfsA rimK ybjN

dusB fis

ycfC purB

purM purN

dcm vsr

def fmt

acrE acrF

rrsE gltV rrlE rrfE

rpsP rimM trmD rplS

relA chpR chpA

fucP fucI fucK fucU fucR

rrsH ileV alaV rrlH rrfH

gadA gadX

serV argV argY argZ argQ

envY ompT

glpF glpK

ibpA ibpB

pflD pflC

baeS baeR

sspA sspB

fabH fabD fabG acpP fabF

rplU rpmA

speF potE

gltB gltD gltF

ompR envZ

fdoG fdoH fdoI fdhE

csgB csgA

argC argB argH

narG narH narJ narI

metT leuW glnU glnW metU glnV glnX

cvpA purF ubiX

argT hisJ hisQ hisM hisP

sdhC sdhD sdhA sdhB b072 b075 sucA sucB sucC sucD

agn4 agn3

flgA flgM flgN

btuC btuE btuD

acs yjcH yjcG

ycbY uup

hsdM hsdS

rtcB rtcA

accB accC

ruvB ruvA

fliL fliM fliN fliO fliP fliQ fliR

potF potG potH potI

rfbB rfbD rfbA rfbC rfbX

ahpC ahpF

rpsL rpsG fusA tufA

sfsA dksA

valV valW

rpoN yhbH ptsN yhbJ ptsO

narZ narY narW narV

hydN hypF

uhpA uhpB uhpC

cobU cobS cobT

csgD csgE csgF csgG

emrR emrA emrB

ybgC tolQ tolR tolA

appC appB appA

mraZ mraW ftsL ftsI murE murF mraY murD ftsW murG murC ddlB ftsQ ftsA ftsZ

artP artI artQ artM artJ

codB codA

hypA hypB hypC hypD hypE

mdoG mdoH

glyW cysT leuZ

aroF tyrA

xapA xapB

hisG hisD hisC hisB hisH hisA hisF hisI

rpsF priB rpsR rplI

mhpA mhpB mhpC mhpD mhpF mhpE

paaA paaB paaC paaD paaE paaF paaG paaH paaI paaJ paaK

tgt yajC secD secF

surA pdxA ksgA apaG apaH

glnH glnP glnQ

kbl tdh

hyfA hyfB hyfC hyfD hyfE hyfF hyfG hyfH hyfI hyfJ hyfR focB

pntA pntB

flhD flhC

rpoZ spoT spoU recG

zraS zraR

tyrT tyrV

chaB chaC

xseB ispA dxs yajO

## S.15 *Escherichia coli* negative (transcriptional boundary) operon examples

yadC panB

proA thrW

metL metF

tufB secE

nusG rplK

acrB ybaJ

prmA yhdG

b4548 glyQ

malM b4559

moaE ybhL

yaiI aroL

aroM yaiE

hflC yjeT

wcaB wcaC

pyrL pyrB

pyrI yjgF

yqgB speA

osmE celA

lacI lacZ

yjbI ubiC

visC gcvT

gcvP ygfF

ybdB cstA

deoD yjjJ

glgX glgC

glgP b3427

alaX b2395

ybdE pheP

phoR brnQ

uup pqiA

pqiB ymbA

trpA yciG

yacC speE

nagD asnB

uxuB uxuR

nrdB yfaE

mrcB fhuA

yjdG dcuB

fumB yjdF

ycaO focA

pflB pflA

ilvN uhpA

betA ykgH

cysH ygcB

yhhU nikA

flhC motA

cheW tar

recF gyrB

flgK flgL

rplT pheM

araD polB

b1007 ycdG

fliT amyA

fecR fecA

fliY b1919

pspE ycjM

ptsP lgt

thyA ppdA

yjjT leuQ

leuV fhuF

hemG rrsA

tehB b1431

rrfC aspT

atoC atoD

atoE atoB

ybcZ nfrB

kup rbsD

rbsK rbsR

treR treB

treC nrdD

yfiA pheL

b1509 hipB

hipA b1506

gor arsR

arsC yhiS

ygbO surE

pcm nlpD

rpmF plsX

fecR fecA

purN ppk

idnR yjgR

thdF tnaL

tnaB yidY

ygaF gabD

radC rpmB

rpmG mutM

menE pmrD

metA aceB

rseC lepA

ttdB ygjE

cadC cadB

cadA yjdL

b2672 nrdH

nrdF proV

araH otsB

trxB cydD

cydC aat

yadN ecpD

htrE yadM

galS mglB

modF galE

galM gpmA

ftsX rpoH

ybfE fldA

ampG cyoA

cyoE yajR

frdD ampC

cysC ygbE

b0024 ribF

lytB yaaF

ychE oppA

ychF b1202

yhfC nirB

cysG yhfL

gidB atpI

atpC glmU

tig clpP

clpX lon

recJ prfB

b4162 glyV

kdpC kdpD

dcd asmA

uraA b2496

cysK ptsH

ybcZ nfrB

nfrA ybcH

dgkA lexA

dinF yjbJ

murI rrsB

rrfB murB

rhaD yiiL

minD minE

minC minD

bioD uvrB

tolA tolB

ybgF lysT

yjdB basR

tsf pyrH

rpsQ rplN

rpmJ rpsM

pinO rpsJ

rpsQ rplN

malY add

malG xylE

yibF selA

selB yiaY

yhdA mreB

cafA tldD

hycH hycI

b2426 cysP

nrfG gltP

leuO ilvI

ilvH fruL

mdlB glnK

rseC lepA

hemY aslA

hofQ aroK

trpS yhfZ

alkB yojI

mukB ycbB

thrC b0005

yjaE thiC

purB phoP

phoQ ycfD

yadB pcnB

folK yadN

nlpB purC

proK dppA

pcm nlpD

glmS pstS

phoU bglG

b0762 modA

serS dmsA

hflB b3177

topB b1762

yihD yihE

dsbA b3861

yjjB dnaT

dnaC yjjA

fixX yaaU

fruA yeiC

gltL ybeK

gntR gntK

fdx yfhJ

zwf edd

ycaP serC

aroA ycaL

hyaF appC

potD ymfA

gloA rnt

ybgF lysT

lysW valZ

xylB yiaB

yggC epd

pgk fba

pheM pheS

himA btuC

rnpA yidD

ybgG cydA

cydB b4513

ybfD ybgA

yeaH yeaI

sppA ansA

fliK fliL

rplA rplJ

rpoC htrC

ycaL cmk

rpsA himD

glpC yfaD

ebgC ygjI

b2097 gatY

yifK argX

proM aslB

kdpF kdpA

kdpC kdpD

rpmJ rpsM

rplQ yhdN

b3007 exbB

yaiB phoA

psiF yaiC

b3407 feoA

feoB yhgG

clpB rrsG

rrfG kgtP

dapB carA

yhcM rplM

rpsI sspA

atpC glmU

glmS pstS

phnB phnC

phnP phnQ

ybbF purE

dnaJ yi811

hdeB yhiD

pqqL gadB

thrT tufB

ftsN hslV

hslU menA

ygdH sdaC

sdaB b2798

ybjN potF

fis b3262

ycfB ycfC

purB phoP

purN ppk

yedJ dcm

fmt sun

acrF yhdV

rrfE yjaA

ffh rpsP

ygcA relA

chpA mazG

yaeD rrsH

rrfH aspU

yhjA gadA

csrA serV

argQ yqaB

ybcH envY

glpK glpX

ibpB yidE

frwB pflD

pflC frwD

yegB baeS

baeR b2080

rpsI sspA

plsX fabH

fabF pabC

rpmA yhbE

potE ybfH

gltF yhcA

b4557 fdoG

csgA csgC

argH oxyR

narK narG

narI b1228

asnB metT

dedD cvpA

ubiX argT

ubiX argT

hisP yfcI

himA btuC

btuD nlpC

yjcG yjcF

uup pqiA

hsdR hsdM

hsdS yjiW

accC yhdT

ruvA ruvB

ruvA ruvB

fliK fliL

fliR rcsA

ybjN potF

potI ybjO

galF rfbB

rfbX glf

yheL rpsL

tufA yheB

yadP sfsA

dksA yadB

valW b1667

yhbG rpoN

ptsO yrbL

narU narZ

narV yddE

ascG hydN

ilvN uhpA

uhpC uhpT

trs56 cobU

cobT erfK

b4518 csgD

ygaH emrR

ybgE ybgC

tolA tolB

hyaF appC

ftsZ lpxC

ybjP artP

prpE codB

hypE fhlA

mdoH yceK

pgsA glyW

leuZ yecA

xapB xapR

hisL hisG

mhpE mhpT

queA tgt

secF yajD

imp surA

dps glnH

glnQ ybiO

htrL kbl

tdh yibD

flhC motA

gmk rpoZ

purU tyrT

tyrV tpr

## S.16 *Escherichia coli* negative (directional) operon examples

b0011 htgA yaaI

htgA yaaI dnaK

carB yaaV caiF

yaaV caiF caiE

yabN yabM leuD

yacE guaC hofC

ppdD b4500 nadC

b4500 nadC ampD

ampE aroP pdhR

lpdA yacH acnB

yacC yacK gcd

yacK gcd hpt

gcd hpt yadF

hpt yadF yadG

yadE panD yadD

panD yadD panC

fhuB hemL yadQ

yafB yafC yafD

gloB yafS rnhA

yafS rnhA dnaQ

yafV ykfE yafH

ykfE yafH gmhA

yafM fhiA mbhA

prfH pepD gpt

crl phoE proB

ykfC trs51 ykfD

insA3 yagJ yagK

yagT yagU ykgJ

ykgK ykgL b4506

ykgB b0302 ykgI

yahM yahN yahO

yahN yahO prpR

yahO prpR prpB

prpR prpB b0332

prpB b0332 prpC

codA cynR cynT

b0370 yaiT tra51

yaiV yaiH sbmA

yaiW b0379 b0380

b0379 b0380 ddlA

b0380 ddlA yaiB

yaiC proC yaiI

b0392 yaiD yajF

malZ yajB queA

xseB yajK thiJ

apbA yajQ yajR

amtB tesB ybaY

tesB ybaY ybaZ

ybaY ybaZ ffs

hemH ybaC gsk

ybaC gsk ybaL

fsr ushA ybaK

ybaP ybaQ ybaR

ybaQ ybaR ybaS

ppiB cysS ybcI

sfmF fimZ argU

fimZ argU intD

argU intD b0538

intD b0538 b0539

b0538 b0539 b0540

ybcX ybcY ylcE

fepD ybdA fepB

ybdA fepB entC

ybdD ybdH ybdL

ybdH ybdL ybdM

ahpF ybdQ ybdR

citB dcuC crcA

cspE crcB ybeH

leuS ybeL ybeQ

ybeL ybeQ ybeR

ybeS ybeT ybeU

yleA yleB glnX

speF ybfK kdpE

phrB ybgH ybgI

sucD farR hrsA

b0753 aroG gpmA

modC ybhA ybhE

ybhA ybhE ybhD

ybhE ybhD ybhH

uvrB ybhK moaA

ybhP ybhQ ybhR

ybiH rhlE ybiA

rhlE ybiA dinG

ybiM ybiN ybiO

ybiF ompX ybiP

ompX ybiP b0816

ybiR ybiS ybiT

ybiS ybiT ybiU

ybiY mipB moeB

b0834 yliG b0836

yliI yliJ dacC

yliJ dacC deoR

ybjG cmr ybjH

b0845 b0846 b0847

b0846 b0847 ybjM

b0847 ybjM grxA

ybjM grxA ybjC

aqpZ ybjD ybjX

ybjD ybjX b0878

ybjZ cspD yljA

dmsC ycaC ycaD

b0899 ycaN ycaK

ycaN ycaK pflA

b0919 ycbC smtA

pncB pepN ycbE

ycbW b0947 ycbY

b0955 ycbG ompA

sulA b0959 b0960

yccF helD mgsA

b0964 b0965 yccV

b0967 b0968 yccK

insB4 cspH cspG

torS torT torR

torT torR torC

b1012 ycdC putA

ycdC putA putP

ycdS ycdT tra53

ycdU serX ycdW

ymdC ymdD mdoG

htrB yceA yceI

flgL rne b1085

yceC yceF yceD

ptsG fhuE ycfF

ycfJ ycfQ ycfR

ycfQ ycfR ycfS

potA pepT ycfD

ymfC icdA ymfD

ymfE lit intE

ymfH ymfI ymfJ

b1172 b1173 minE

minC ycgJ ycgK

ycgJ ycgK ycgL

ycgN hlyE umuD

nhaB fadR ycgB

fadR ycgB dadA

b1192 mltE ycgR

mltE ycgR ymgE

b1200 b1201 b1202

pth ychH ychM

chaC ychN ychP

ychN ychP narL

galU hns tdk

hns tdk ychG

yciI tonB yciA

yciC yciD yciE

yciK sohB yciN

sohB yciN topA

acnA ribA pgpB

goaG pspF pspA

ompG ycjW ycjX

tyrR tpx ycjG

tpx ycjG ycjI

b1329 b1330 trs54

ydaL b1341 b1342

ydaD sieB ydaF

b1369 trs55 b1371

ydbL feaR feaB

feaR feaB tynA

ynbD acpD hrpA

ydcA b1420 trg

b1420 trg b1422

trg b1422 b1423

ydcG b1425 ydcH

rimL b1428 tehA

b1432 b1433 ydcN

b1450 b1451 b1452

b1451 b1452 ansP

b1452 ansP b1454

ydcE b1462 b1463

b1462 b1463 yddE

b1481 osmC b1483

ydeA ydeB marR

marB ydeD ydeF

ydeD ydeF ydeH

ydeF ydeH ydeG

ydeH ydeG ydeI

ydeG ydeI ydeJ

ydeI ydeJ dcp

ydeJ dcp ydfG

nohA ydfO b1550

cspB cspF b1559

speG ynfC b1586

b1600 b1601 pntB

ydgB ydgC rstA

add b1624 b1625

b1640 slyB slyA

slyB slyA b1643

lhr ydhD ydhO

sodB b1657 purR

b1657 purR ydhB

purR ydhB ydhC

cfa ribE ydhE

ribE ydhE b1664

ydhE b1664 valV

b1695 b1696 ydiQ

ydiD ppsA ydiA

thrS b1721m b1722

b1721m b1722 pfkB

b1725 b1726 yniC

b1731 katE ydjC

b1757 b1758 b1759

b1758 b1759 b1760

b1759 b1760 gdhA

b1760 gdhA b1762

yeaK b1788 yeaL

b1788 yeaL yeaM

yeaL yeaM yeaN

yeaO yoaF yeaP

yoaF yeaP yeaQ

b1810 b1811 pabB

b1815 yoaE manX

b1826 b4526 b1827

b4526 b1827 b1828

b1827 b1828 htpX

yebG purT eda

ruvA yebB ruvC

insA5 yecG otsA

ftn yecH tyrP

yecH tyrP yecA

uvrY yecF sdiA

amyA yedD yedE

yedM b1936 fliE

b1936 fliE fliF

rcsA dsrB b1953

dsrB b1953 dsrA

b1953 dsrA b1955

dsrA b1955 b1956

yedI yedA vsr

b1974 serU b1976

yeeO asnU cbl

nac asnV erfK

trs56 b1995 yi223

dacD sbcB yeeD

wza b2063 asmA

udk yegE alkA

yegE alkA b2069

alkA b2069 b2070

b2069 b2070 b2071

b2070 b2071 b2072

bglX dld pbpG

yohC yohD yohF

folE yeiG cirA

yeiK b2163 yeiM

rplY yejK yejL

proL yejO b2191

yejO b2191 trs58

b2191 trs58 narP

trs58 narP ccmH

napF eco yojH

ada b4532 yojL

rcsB rcsC atoS

gyrA ubiG yfaL

ubiG yfaL nrdA

yfaE inaA yfaH

inaA yfaH glpQ

b2250 yfaO ais

yfaO ais b2253

elaD yfbK yfbL

yfbM yfbN b2274

pdxB div b2322

fabB b2324 b2325

b2330 b2331 b2332

b2345 vacJ yfdC

b4535 dsdC dsdX

b2375 b2376 b2377

b2376 b2377 b2378

b2377 b2378 b2379

b2378 b2379 b2380

b2391 b2392 nupC

yfeD gltX valU

xapA yfeN yfeR

yfeN yfeR yfeH

yfeR yfeH b4536

crr pdxK yfeK

focB perM b2494

guaB xseA b2510

b2520 b2521 b2522

yphG b2550 glyA

b2550 glyA hmpA

glyA hmpA glnB

purL yfhD yfhC

rnc b4539 lepB

rpoE nadB yfiC

nadB yfiC srmB

yfiC srmB b2577

srmB b2577 yfiK

b2577 yfiK yfiD

yfiK yfiD ung

yfiD ung yfiF

ung yfiF trxC

b4540 grpE yfjB

intA yfjH alpA

b2650 b2651 ileY

b2651 ileY b2654

ygaP stpA b2670

stpA b2670 ygaC

b2670 ygaC b2672

alaS b4542 oraA

b2708 ygaA b2710

pphB ygbI b2736

cysD iap ygbF

ygcE ygcF b2778

ygcF b2778 eno

ygcA barA ygcX

metV b2817 argA

b2817 argA recD

aas galR lysA

galR lysA lysR

lysA lysR ygeA

b2848 yqeK ygeF

b2868 ygeV ygeW

xerD fldB b2896

ygfY ygfZ b2899

yqfB bglA ygfF

tktA yggG speB

yqgB yqgC yqgD

yqgC yqgD metK

yqgF yggR yggS

b2964 b2965 yqgA

glcD glcC b2981

glcC b2981 trs59

b2981 trs59 b2983

yghS yghT pitB

gsp b2989 hybG

b2998 b3001 yqhA

b3001 yqhA yghA

yghB yqhC yqhD

qseC b3027 mdaB

ygiC ygiD ygiE

ygiD ygiE b4544

yqiI glgS b3050

ygiG ygiH ygiP

ygiH ygiP ttdA

ygjE ygjD rpsU

rpoD ygjF ileX

ygjF ileX yqjH

ileX yqjH yqjI

yqjH yqjI aer

yqjI aer b3073

aer b3073 ygjH

b3073 ygjH ebgR

yhaI yhaJ yhaK

yhaV agaR agaZ

yraK yraL yraM

yraR yhbO yhbP

yhbO yhbP yhbQ

yhbP yhbQ yhbS

metY argG b3173

ftsJ yhbY b3181

yhbY b3181 dacB

b3181 dacB yhbZ

yhcE trs510 b4545

b3226 yhcL sspB

degS mdh argR

yhcR qseA tldD

yhdA yhdH b3254

yhdH b3254 accB

yhdU envR acrE

rrsD b3279 yrdB

fkpA slyX slyD

prkB yhfA crp

b3395 mrcA yrfE

mrcA yrfE yrfF

yrfI yhgE pckA

yhgE pckA envZ

yhgA bioH yhgH

malP malT yhgKJ

rtcB rtcR glpR

glpE glpD b3427

asd yhgN gntK

yrhB ggt yhhA

ggt yhhA ugpQ

livK yhhK b3460

yhhL yhhM yhhN

zntA yhhP yhhQ

b3472 yhhS b3474

yhiL yhiM yhiN

yhiM yhiN pitA

yhiN pitA yhiO

pitA yhiO uspA

yhiS trs511 b3506

yhjA treF yhjB

treF yhjB yhjC

yhjH kdgK yhjJ

yiaC bisC yiaD

yiaE yiaF yiaG

cspA yiaZ yi5A

b4548 yiaH yiaA

xylR bax malS

sgbE yiaT yiaU

yiaT yiaU yiaV

mtlR b4550 yibL

b3646 yicF gmk

recG gltS yicE

yicL nlpA b4552

nlpA b4552 yicM

yicO yicP uhpT

ivbL emrD yidF

yidH yidI yidJ

yidK yidL glvG

b4553 yidP yidE

ibpA yidQ yidR

yidQ yidR yidS

yidR yidS b3691

yidW yidX yidA

yieF yieG yieH

asnC asnA yieM

trpT yifDA yifE

yifDA yifE yifB

yifE yifB ilvL

ilvA ilvY ilvC

ilvY ilvC ppiC

ilvC ppiC rep

ppiC rep gppA

hemC cyaA cyaY

cyaA cyaY b3808

uvrD b4566 corA

b4566 corA yigF

yigM metR metE

metR metE ysgA

metE ysgA udp

b4556 rfaH yigC

b3861 yihG polA

spf yihA yihI

rhaR rhaT b3908

glpF yiiU menG

priA rpmE yiiX

ptsA yijI b4558

yijI b4558 frwC

oxyR udhA yijC

yijD trmA btuB

yjaH yjaI hydH

yjaA yjaB metA

yjbB pepE yjbC

pepE yjbC yjbD

ubiA plsB dgkA

yjbJ b4046 yjbL

b4050 qor dnaB

yjbR uvrA ssb

uvrA ssb b4060

ssb b4060 yjcC

b4060 yjcC soxS

yjcC soxS soxR

b4089 rpiB phnQ

b4140 yjeH mopB

glyY yjeS yjeF

b4189 yjfP yjfQ

sgaE yjfY rpsF

rplI yjfZ ytfA

yjfZ ytfA ytfB

ytfA ytfB b4207

ytfG b4212 cpdB

b4212 cpdB cysQ

ytfI ytfJ ytfK

ytfJ ytfK ytfL

ytfP yjfA chpS

chpB ppa ytfQ

yjfF fbp yjfG

fbp yjfG yjgA

yjfG yjgA pmbA

treR mgtA yjgF

pyrL yjgG yjgH

yjgL argI yjgD

argI yjgD b4256

yjgD b4256 yjgN

b4256 yjgN valS

idnD idnK b4269

idnK b4269 leuX

yjgY yjgZ yi41

yjgZ yi41 b4279

yjhC yjhD yjhE

fecI insA7 yjhU

yjhQ yjhR yjhS

fimH gntP uxuA

uxuR b4325 b4326

b4325 b4326 yjiE

yjiH b4331 yjiJ

yjiQ yjiR yjiS

hsdR mrr yjiA

yjiY tsr yjiZ

yjjM yjjN mdoB

nadR yjjK slt

trpR yjjX gpmB

yjjX gpmB rob

gpmB rob creA

creD arcA yjjY
