# Supplementary material for: Predicted transcription factor binding sites as predictors of operons in Escherichia coli and Streptomyces coelicolor
Source: BMC Genomics. 2008 Feb 12;9:79. doi: 10.1186/1471-2164-9-79 (PMC2276206; doi:10.1186/1471-2164-9-79)
Supplement: Additional file 2 — Repressor and Activator site footprint data. This file contains full details of all the 211 repressor sites, 77 activator sites and 341 dual regulator sites taken footprint data in RegulonDB for E. coli and used in this study for analysing trends in palindromicity. [file 1471-2164-9-79-S2.doc]

# Additional data file 2

## S.2.1 Escherichia coli Repressor footprint data

| Repressor | Footprint sequence (uppercase denotes the actual footprint) |
| --- | --- |
| MarR | tcatttgactTATACTTGCCTGGGCAATATTATcccctgcaac |
| MarR | cctgcaactaATTACTTGCCAGGGCAACTAATGtgaaaagtac |
| DeoR | cccgtcatttTGTTACTCTGCTTACAtcacctggat |
| DeoR | cgcgcggcaaTCCCTTCTTTTTCACAcagcaagtta |
| DeoR | gtagccccctTTTGTGAAAATTTTATcatgcaccgg |
| DeoR | atctcgtcttGTGTTAGAATTCTAACatacggttgc |
| DeoR | gcaggaagctGTGGGTAAGGCAGATTgttttctgct |
| DeoR | attgttttctGCTTCCAGTGCCAGAAaatggcgctt |
| DeoR | tgcggagtagATGTTAGAATACTAACaaactcgcaa |
| Mlc | ccgttgaatgAGTTTTTTTAAAGCTCGTAATTAATGgctaaaacga |
| Mlc | attggcactgAATTATTTTACTCTGTGTAATAAATAaagggcgctt |
| Mlc | atagcctacaGATTATTTCGGAGCGCGAAAATATAGggagtatgcg |
| Mlc | atagcctacaGATTATTTCGGAGCGCGAAAATATAGggagtatgcg |
| Mlc | aatgtgacaaGGATATTTTACCTTTCGAAATTTCTGctaatcgaaa |
| Mlc | cacataaaatAATTTTTTCGATATCTAAAATAAATCgcgaaacgca |
| Mlc | ttaaccagacTAATTATTTTGATGCGCGAAATTAATcgttacagga |
| Mlc | gaccttataaCCATTAATTACGAAGCGCAAAAAAAAtaatatttcc |
| Fur | ttacgctattGCAAATGCAAATAGTTATCaataatatta |
| Fur | gataatattaTTGATAACTATTTGCATTTgcaatagcgt |
| Fur | gcgtggcaatTCTATAATGATACGCATTAtctcaagagc |
| Fur | gcgtggcaatTCTATAATGATACGCATTAtctcaagagc |
| Fur | gcaatataatGAGAATTATTATCATTAAAagatgatttg |
| Fur | gtcgcggcaaTATAATGAGAATTATTATCattaaaagat |
| Fur | taatgcttatCAAAATTATTATCACTTTCacgagcacta |
| Fur | aagtgataatGCTTATCAAAATTATTATCactttcacga |
| Fur | cgctccaagtGATAATGCTTATCAAAATTattatcactt |
| Fur | gaaaatcgctCCAAGTGATAATGCTTATCaaaattatta |
| Fur | catttgcattTACAAACAAAATTATTCGCacactaaata |
| Fur | ttgccttattGAATATGATTGCTATTTGCatttaaaatc |
| Fur | gtttttctacTGAAATGATTATGACTTCAatgacccttg |
| Fur | aattgttatcGTTTGCATTATCGTTACGCcgcaatcaaa |
| Fur | attgataattGTTATCGTTTGCATTATCGttacgccgca |
| Fur | atataaaattTAACATTTGGATTGATAATtgttatcgtt |
| Fur | atgcggatatAAAATTTAACATTTGGATTgataattgtt |
| Fur | catatccaggGTAATTTCGACCACTATTTgctatatatt |
| Fur | aatatatagcAAATAGTGGTCGAAATTACcctggatatg |
| Fur | ctctgttagcAACGGCAATCGGCCTCGTGgcagcgattc |
| Fur | aatagtaatgAGAACGACTATCAATTCGAcgtcgttttg |
| Fur | aagtgcgaatGAGAATGATTATTATTGTCtcgcgatcag |
| Fur | ttggacaagtGCGAATGAGAATGATTATTattgtctcgc |
| Fur | acgaaaagtaCGGCATTGATAATCATTTTcaatatcatt |
| Fur | gtacggcattGATAATCATTTTCAATATCatttaattaa |
| Fur | acaattttatTTCCAATTGTAATGATAACcattctcata |
| TrpR | tttacaatcgAAATTGTACTAGTTTGATGGTATGATCgctattctca |
| TrpR | gacaattaatCATCGAACTAGTTAACTAGTACGCAAGttcacgtaaa |
| TrpR | tgttgacaatTAATCATCGAACTAGTTAACTAGTACGcaagttcacg |
| TrpR | cttagtcgccGAATGTACTAGAGAACTAGTGCATTAGcttatttttt |
| TrpR | tttttttctgTCTTTTGTACTCGTGTACTGGTACAGTgcaatgcata |
| TrpR | tatgatatgcTATCGTACTCTTTAGCGAGTACAACCGggggaggcat |
| PurR | tcacagccacGCAACCGTTTTCCTTGctctctttcc |
| PurR | aaacaggttcGGAAAACGTTTGCGTTttttttgccg |
| PurR | tttttcgtgcAAAGGAAAACGTTTCCgcttatcctt |
| PurR | agtacgttggCTGCAAAAACTGGCCGcgcacgcagc |
| PurR | aggtgtgtaaAGGCAAACGTTTACCTtgcgattttg |
| PurR | gtagcgaaacGAGCAAACGTTTCCACtacaactgtg |
| PurR | ataaagacacACGCAAACGTTTTCGTttatactgcg |
| PurR | aggaaatcccTACGCAAACGTTTTCTttttctgtta |
| PurR | ctgatatgatACGCAAACGTGTGCGTctgcaggaaa |
| PurR | gtaaagcagtCTCGCAAACGTTTGCTttccctgtta |
| PurR | aaaggggtagATGCAATCGGTTACGCtctgtataat |
| PurR | aaaggaatttACGCAAACGATTACCTtcaggctacg |
| PurR | gcgtagcctgAAGGTAATCGTTTGCGtaaattcctt |
| PurR | cacgagctggATGCAAACGATTTCAAggaatgaatt |
| PurR | cacgagctggATGCAAACGATTTCAAggaatgaatt |
| PurR | tttatttccaCGCAAACGGTTTCGTCagcgcatcag |
| PurR | gtttcggtggTAAGTTCAGGCAAAAGagaacgattg |
| PurR | gatgaggatgTGGCAAACGTTTGCTTttctctttcg |
| PurR | cgcgagcgttGCGCAAACGTTTTCGTtacaatgcgg |
| BetI | aaagcggttaTTGATTGGACGTTCAATATAAaatgtgtctt |
| BetI | ttaagacacaTTTTATATTGAACGTCCAATCaataaccgct |
| YiaJ | agattgccttGTTAAAAAGTGATCGATATATTTGAAATCAAGTTTcgcatattga |
| MalI | aataaaaggtAAAACGTTTTATctgtcacata |
| MalI | atgtgacagaTAAAACGTTTTAccttttattt |
| MalI | gcgagaagatAAAACGTTTTATcaaattttag |
| MalI | aaaatttgatAAAACGTTTTATcttctcgcgc |
| ArgR | acgtcatcatTGTGAATTAATATGCAaataaagtga |
| ArgR | tgcaaataaaGTGAGTGAATATTCTCtggagggtgt |
| ArgR | cacatataaaGTGAATTTTAATTCAAtaagtggcgt |
| ArgR | ggggttgcaaATGAATAATTACACATataaagtgaa |
| ArgR | cctgcgaaaaAACAGAATAAAAATACactaatttcg |
| ArgR | gattattcgaAATTAGTGTATTTTTAttctgttttt |
| ArgR | aatacactaaTTTCGAATAATCATGCaaagaggtgt |
| ArgR | attgctaatcATGTGAATGAATATCCagttcacttt |
| ArgR | aaagatgattAAATGAAAACTCATTTattttgcata |
| ArgR | aactcatttaTTTTGCATAAAAATTCagtgagagcg |
| ArgR | agggctgactGTTTGCATAAAAATTCatctgtatgc |
| ArgR | attcatctgtATGCACAATAATGTTGtatcaaccac |
| ArgR | atgcatatttTGTGGTTATAATTTCAcatttgttta |
| ArgR | attgccatttAGTGATTTTTTATGCAtattttgtgg |
| ArgR | gtccaacactTTTTGCAAAAAAATGCatttgactcg |
| ArgR | taaggcaattAAATGAAAAGGAATTTattatcttcc |
| ArgR | tcatgatagtATCAATATTCATGCAGtatttatgaa |
| ArgR | tatttttattCATAAATACTGCATGAatattgatac |
| ArgR | atgcagtattTATGAATAAAAATACActaacgttga |
| ArgR | tattacgctcAACGTTAGTGTATTTTtattcataaa |
| ArgR | tccatataaaTTGAATTTTAATTCATtgaggcgtta |
| ArgR | agacttgcaaATGAATAATCATCCATataaattgaa |
| MetJ | caaaaatggaTAGAGGTGcaatggatat |
| MetJ | ttagacgtctGGATGCCTtaacatccat |
| MetJ | gttaaggcatCCAGACGTctaaatcaat |
| MetJ | tgattgatttAGACGTCTggatgcctta |
| MetJ | gaagatgatcCAGACGTTtacccagccc |
| MetJ | gagtgatcgaGTTAACATtgttaagtta |
| MetJ | gtggcaaaatCTGGATGActattacaac |
| MetJ | tatgaaattcCTGCATCTttattgacct |
| MetJ | caacagcagcAAAGAAATttatcagtac |
| MetJ | ccaggatgaaTAAACATTgttcatggca |
| MetJ | atccataagaAAGGTCAGgcacactggg |
| MetJ | tcatcggagtGTAAACATcctgctattt |
| MetJ | tttatacgtcTGGATGTCtaaactagca |
| MetJ | gtttagacatCCAGACGTataaaaacag |
| MetJ | atcgtctggaTAGATGTGaatgctaaag |
| MetJ | gtgtaaacatCTGGACGGctaaaatcct |
| MetJ | tcaatacatcTGGACATCtaaacttctt |
| MetJ | ttcggcttttCCTTCATCtttacatctg |
| MetJ | ttcatctttaCATCTGGAcgtctaaacg |
| MetJ | gacgtctaaaCGGATAGAtgtgcacaac |
| MetJ | taaatactgaAATGTCCGgcagacaagt |
| MetJ | atcttcagctATCTGGATgtctaaacgt |
| MetJ | atgtctaaacGTATAAGCgtatgtagtg |
| LexA | ggcagtaatgACTGTATAAAACCACAGCCAatcaaacgaa |
| LexA | agggttgacaTCCGTTTTTGTATCCAGTAActctaaaagc |
| LexA | tacaattcaaGCTGAATAAATATACAGCACaggagatacc |
| LexA | ttatcctgacGCCTGGCTTTCAGGGCAGCGttatttcgaa |
| LexA | ttatggtgatGAACTGTTTTTTTATCCAGTataatttgtt |
| LexA | ttgattacacTCCTGTTAATCCATACAGCAacagtactgg |
| LexA | ctggatgtacTGTACATCCATACAGTAACTcacaggggct |
| LexA | ggttattttaCCTGTATAAATAACCAGTATattcaacagg |
| LexA | gcatctccagACCTGTTTAAATATCCAGTAttgaggcaag |
| LexA | ctgaagttatACTGTTTTTATATACAGTAGtctgttcttg |
| LexA | aagaacagacTACTGTATATAAAAACAGTAtaacttcagg |
| LexA | catgagtggcCACTGGTTATCTTTACCGTAttgggtcaat |
| LexA | aaagaaatatACTGTATAAAATCACAGTTAttatgagagg |
| LexA | ctctcataatAACTGTGATTTTATACAGTAtatttctttt |
| LexA | ttgcaactatACTGTATATAAAAACAGTATcaatggaggc |
| LexA | ccagcctcttTACTGTATATAAAACCAGTTtatactgtac |
| LexA | aaccagtttaTACTGTACACAATAACAGTAatggtttttc |
| LexA | aaacacttgaTACTGTATGAGCATACAGTAtaattgcttc |
| LexA | cgatcgcggcAACTTTATGTACAGCCAGTGgaatggcggt |
| LexA | attttgaaatAAGCTGGCGTTGATGCCAGCggcaaaccga |
| LexA | agatactcacAACTGTATATAAATACAGTTacagatttac |
| LexA | atagaagtttACTGTATAAATAAACAGTAAtatttggaca |
| LexA | taatcagcaaATCTGTATATATACCCAGCTttttggcgga |
| LexA | ctaaggcaggTCTGAATGAATACCCAGTATaattccagtt |
| LexA | aaatcgccttTTGCTGTATATACTCACAGCataactgtat |
| LexA | atactcacagCATAACTGTATATACACCCAgggggcggaa |
| LexA | caggtcaattTGTGTCATAATTAACCGTTTgtgatcgccg |
| LexA | tgacacaaatTGACCTGAATGAATATACAGtattggaatg |
| LexA | atgcattccaATACTGTATATTCATTCAGGtcaatttgtg |
| GlpR | atatccaaatAAGATCGATAACGATAATTAatttcattat |
| GlpR | ggacattttcATGAATCGTATTGAACATTAtcatgactgg |
| GlpR | gggtttaataAACTTCGATTTTTAGCAATTagtgctattc |
| GlpR | agatttaaatATGTTCAAAGTGAAGAATGAattatgacaa |
| GlpR | tgtcataattCATTCTTCACTTTGAACATAtttaaatctt |
| GlpR | aaagctgcgtCATGCGTGATATGCAGCATTttcgcaagat |
| GlpR | aaaacatgatTAAGGTCAAAAATGTTTGATatttacaatt |
| GlpR | ataatgtgtgCGGCAATTCACATTTAATTTatgaatgttt |
| GlpR | aagatttacaAAATGTTCAAAATGACGCATgaaatcacgt |
| GlpR | aacgtgatttCATGCGTCATTTTGAACATTttgtaaatct |
| GlpR | atcacgtttcACTTTCGAATTATGAGCGAAtatgcgcgaa |
| GlpR | ttcgcgcataTTCGCTCATAATTCGAAAGTgaaacgtgat |
| GlpR | tatgagcgaaTATGCGCGAAATCAAACAATtcatgttttt |
| GlpR | aaaaacatgaATTGTTTGATTTCGCGCATAttcgctcata |
| GlpR | actatggctaAATGGTAAAAAACGAACTTCagagggataa |
| GlpR | ttatccctctGAAGTTCGTTTTTTACCATTtagccatagt |
| GlpR | tttcacgctaTTTGTTGGAGAACAAACATTtattttatca |
| GlpR | aagctcataaATGTTCGTTATCGAACATATtagcaaagaa |
| GlpR | tctttgctaaTATGTTCGATAACGAACATTtatgagcttt |
| GlpR | actttcgttaAAGCTCATAAATGTTCGTTAtcgaacatat |
| GlpR | taacgaacatTTATGAGCTTTAACGAAAGTgaatgagggc |
| GlpR | gggatcaactGGTTTGCGTTTTGGCGCAAAttcagtgtta |
| GlpR | aaccggaaatTAAGCGCGGATTCGAATATTctgactgttg |
| GlpR | ttagcgtgtcGCAGCGCGAATTTGAGCAAAtctacccaaa |
| GlpR | ccagctcccgCGCGGTCGTAATGGATCACGatgccaatat |
| GlpR | atttctcgttTTTGCTCGTTAACGATAAGTttacagcatg |
| GlpR | acacacatttTAAGTTCGATATTTCTCGTTtttgctcgtt |
| GlpR | acgctcatttTATGACGAGGCACACACATTttaagttcga |
| GlpR | gatggaataaATGGCGCGATAACGCTCATTttatgacgag |
| BirA | tgtcataatcGACTTGTAAACCAAATTGAAAAGATTTAGGTTTACAAGTCtacaccgaat |
| BirA | aattcggtgtAGACTTGTAAACCTAAATCTTTTCAATTTGGTTTACAAGTcgattatgac |
| LctR | tacacaacacAATTGGCAGTGCCACTTttacacaacg |
| LacI | gaatccgacgGGTTGTTACTCGCTCACATTtaatgttgat |
| LacI | atgttgtgtgGAATTGTGAGCGGATAACAAtttcacacag |
| LacI | actggaaagcGGGCAGTGAGCGCAACGCAAttaatgtgag |
| GcvA | gctaaattaaCACATATTTAATTAACTAATAGGTATTGCtatgtcaact |
| GcvA | tttataatggCTCAGATTAAAAAAACTAATAGGTTACATagtgtgatct |
| GcvA | agctcaacggACAATTTATAATGGCTCAGATTAAAAAAActaataggtt |
| GcvA | ttttaaccatAAGCTAATGTGATGATCAATTTTACCTTAtggttaacag |
| GcvA | tttttgcattTTTTAACCATAAGCTAATGTGATGATCAAttttacctta |
| GcvA | tcacctccgtTTTCGCATTATATTTTCTAATGCCATTATtttttgattt |
| GcvA | ggagattttcCTCATTTGAAATAAACTAATTTCACCTCCgttttcgcat |
| GntR | gaatccacaaTTGTTACGCGTAACAAatcgaatcat |
| GntR | tttatggttaTTTTACCGGTAACATGatcttgcgca |
| GntR | caaacgccagATGTTACCCGTATCATtcacatgggt |
| GntR | tcagaaaattGACGTTACCCATAACAaatgaaaggc |
| GalR | gatgactttcCTGTGTAAACGTTACCAattgtttaag |
| GalR | ttatgagagtTCTGGTTACCGGTGGTAgcggttacat |
| GalR | ttatgagagtTCTGGTTACCGGTGGTAgcggttacat |
| GalR | gctaaattctTGTGTAAACGATTCCACtaatttattc |
| GalR | gctaaattctTGTGTAAACGATTCCACtaatttattc |
| GalR | agataaatgtTAGTGTAAGCGATTACActgatgtgat |
| IclR | ttgttgctctCCTGTGGGCTTAAAAAGGAAATTATAGACTACGTgagaaacagc |
| IclR | agtaaccaatAAATGGTATTTAAAATGCAAATTATCAGGCGTACcctgaaacgg |
| IclR | tatcaagtatTTTTAATTAAAATGGAAATTGTTTTTGATTTTGCattttaaatg |
| GalS | ggttttaatcATGGCAACTGTTTCCAtgcgcgacat |
| GalS | gcgctaatgcCTGGCAACGGTTTCGTatcgtcacgc |
| GalS | ggataacacgCTGGCAACGGTTACCTttaaaatcgc |
| GalS | tggcgtctctGTGGCAACGGTTTCCCgggtgctcaa |
| GalS | aacaaaacggCTGTAACCGTTTCCATtgctgtgact |
| GalS | catgaaaattCTGTAACCGTTTTCACgcgctatctg |
| GalS | agtcgaaaaaCGGCAACGGTTTGCCAccatcctgtc |
| MarR | tcatttgactTATACTTGCCTGGGCAATATTATcccctgcaac |

## S.2.2 Escherichia coli Activator footprint data

| Activator | Footprint sequence (uppercase denotes the actual footprint) |
| --- | --- |
| RhaS | aactggatgcCGTCAGTAACGAAGAGCtttccgccat |
| RhaS | aagaaggtagTAGCAACCGGGCAAAGAttgaccagac |
| RhaS | gattttccccAGGCAACTGGGGAAAGAccaaaccggg |
| RhaS | cattatccggTGGCAACCAGCAAAAAAtcctcattgc |
| RhaS | atacacaataCGGCCAACAGAGAAAGAtacctgtgct |
| RhaS | atcatcggcgTCAGTGGCGCGAAGGTAaacatcggcg |
| RhaS | ttcatctatgAAGCAACCAGGGGAAGAactgcaggaa |
| RhaS | cattttcctgTCAGTAACGAGAAGGTCgcgaattcag |
| RhaS | ccattttcctGTCAGTAACGAGAAGGTcgcgaattca |
| RhaS | aatgggccatTGGCAACCAGGGAAAGAtgaacgtgat |
| RhaS | catcacgttcATCTTTCCCTGGTTGCCaatggcccat |
| RhaS | ccgtgattgcCAGTAAATCGACAACGGcggcaacagg |
| RcsB | cagtggcctgATGAATTGAAGATTCATCTGGTTGAatatgtgccg |
| RcsB | ctgatattatTAGTGAATTGCCATTAATATAATTCcgtaacgttt |
| RcsB | taaaaaccatATTGAATGACACTTAATATAATTCTtaaaaatagc |
| MalT | taatcaccggAGACGGGAGGattttttctt |
| MalT | ctatcaaaaaTCGCTCACCCtttttcacct |
| MalT | actggctggaTGATGAAGGTcgcgatcagg |
| MalT | acacattacaTCGCTCACCCtggctgacgg |
| MalT | aatcccccgcAGGATGAGGAaggtcaacat |
| MalT | ttgctcatccCCGCAACTCCtccctgccta |
| MalT | tttgcgcgagATCGCTCACCcttgcttctc |
| MalT | cttgcttctcATCCTGTGGActtaccgctc |
| MalT | cttaccgctcAGGGATGAGTtttgtttggc |
| MalT | cttaccgctcAGGGATGAGTtttgtttggc |
| MalT | agcccgtataATCCTCCACCcggcgcgcca |
| MalT | gtggggcgtaGGGGCAAGGAggatggaaag |
| MalT | ctttccatccTCCTTGCCCCtacgccccac |
| MalT | tttatgggggAGGAGGCGGGaggatgagaa |
| MalT | ttctcatcctCCCGCCTCCTcccccataaa |
| MalT | atggcttaaaTCCTCCACCCcctggctttt |
| MalT | aagccaggggGTGGAGGATTtaagccatct |
| MalT | taaagtggcaTCCTCCCGCAtcctctctga |
| XapR | aatgcgtgagAAAAGTGTATTGGtaagagccgg |
| XapR | ggaattaataTCGCCAATACAGTttttaccgtg |
| FecI | cttctcgttcGACTCATagctgaacac |
| KdpE | ccatttttatACTTTTTTTACAccccgcccgc |
| KdpE | ccatttttatACTTTTTTTACAccccgcccgc |
| KdpE | cgttttttatTCTTTTTATTCAcccacatcgc |
| TdcA | tgatgttatcGCCATAAAATATGGTtatccccgtc |
| XylR | ctcaatagcaGTGTGAAATAACATAAttgagcaact |
| XylR | agcgcacactTGTGAATTATCTCAATagcagtgtga |
| XylR | cataaatcaaGAAATAAACCAAAAATcgtaatcgaa |
| XylR | aaaaatcgtaATCGAAAGATAAAAATctgtaattgt |
| CaiF | acatctgactTTCAATATTGGTGATCCATAAAACAATATTGAAAATTTCTTTTTGctacgccgtg |
| CaiF | acacggcgtaGCAAAAAGAAATTTTCAATATTGTTTTATGGATCACCAATATTGAaagtcagatg |
| CaiF | tgctacgccgTGTTTTCAATATTGGTGAGGAACTTAACAATATTGAAAGTTGGATttatctgcgt |
| CaiF | cacgcagataAATCCAACTTTCAATATTGTTAAGTTCCTCACCAATATTGAAAACacggcgtagc |
| Cbl | ttgttagaacGGAGTAATTGCATATTTAATCTTTCCTTAGCCGTTTTTTTGCTAAgaataaaatc |
| UhpA | tttccaggttTTGCCTGGACGCTATCTCAGGCCTgatttgctgc |
| UhpA | cacttcatatCCGGCAAAACTAAGAAATTTTCCAGGTTTTGCCTGGACgctatctcag |
| UhpA | catatccggcAAAACTAAGAAATTTTCCAGGTTTtgcctggacg |
| FhlA | gctttcccctGCGACACGGGTGTCGAatccattttt |
| FhlA | attgtcatttTTGACGAAAACATGCCGATGAAATGTCATTTTCGACACTCatcgacacgc |
| FhlA | ccctgcggtgAATAATGTCGATGATGTCGAAATGACACGTCGACACGGCGacgaaattca |
| FhlA | ctgtcacgcgGTATTTCGTTTCGTCACGTCAAAACTGACGACAGCCTGTTtttcgtcaga |
| IciA | gcttagcattTAACAATAACCGAatagaaaaca |
| IciA | ttatatattgACCACAACTGATAcatcagatta |
| IciA | aaaaagattaATAAGCCATCTATatcaatttat |
| IciA | atggtcattaAATTTTCCAATATgcggcgtaaa |
| NarP | gctacaggttTTACCCCGATCGGGGTATGcatctttgac |
| MarA | tgcgcttcttGTTTGGTTTTTCGTGCCAtatgttcgtg |
| MarA | ttcatcgggcTATTTAACCGTTAGTGCCTCCtttctctccc |
| MarA | ccgccgcggcAAAAGCAGAAACTGTAAAACGcagcagtagc |
| MarA | aacttgaaccGATTTAGCAAAACGTGGCATCggtcaattca |
| MarA | cttttcccgtAATCGCACGGGTGGATAAGCGtttacagttt |
| MarA | ttcaaagcgtCATCGCATAAACCACTACATCttgctcctgt |
| MarA | ttcattaataCGACACGTTTCATTAAGAttttcctcag |
| MarA | ttacgaaaagTACGGCATTGATAATCATTTTcaatatcatt |
| MarA | cctctgattgATTTGATCGATTGAGCCTTCCagtccttcgg |
| TdcR | ttaaccacatAACGAATGATGTtatcgccata |
| CspA | agtatatcagGCATTggatgtgaat |
| CspA | cttctactccGTAATtggcaagaca |
| CspA | tttttcggcaTTCATtggcacttct |
| RhaR | aaattcacgcTGTATCTTGAAAAATCGACGttttttacgt |
| RhaR | aaaaatcgacGTTTTTTACGTGGTTTTCCGtcgaaaattt |
| RhaR | gtggttttccGTCGAAAATTTAAGGTAAGAacctgacctc |
| RhaS | aactggatgcCGTCAGTAACGAAGAGCtttccgccat |

## S.2.3 Escherichia coli Dual regulator footprint data

| Dual regulator | Footprint sequence (uppercase denotes the actual footprint) |
| --- | --- |
| CytR | aacgcaatcgATTACGTAAATGATAGAACTGTGAAACGAAACATATTTTTgtgagcaatg |
| CytR | acgtgaacgcAATCGATTACGTAAATGATAGAACTGTGAAACGAAACATAtttttgtgag |
| CytR | gcgtcgcgcaTTTTTGATGTATGTTTCACGCGTTGCATAATTAATGAGATtcagatcaca |
| CytR | tccacatcacAATTTCGTTTTGCAAATTGGGAATGTTTGCAATTATTTGCcacaggtaac |
| CytR | tctgtttaaaTGTTTTATTGCAATCGGTTGCTAAATTGCATTTTAAGAGGtgattttgat |
| CytR | tctgtttaaaTGTTTTATTGCAATCGGTTGCTAAATTGCATTTTAAGAGGtgattttgat |
| CytR | tctgtttaaaTGTTTTATTGCAATCGGTTGCTAAATTGCATTTTAAGAGGtgattttgat |
| CytR | atgtcacattTTGTGCGTAATTTATTCACAAGCTTGCATTGAACTTGTGGataaaatcac |
| CytR | atgtcacattTTGTGCGTAATTTATTCACAAGCTTGCATTGAACTTGTGGataaaatcac |
| CytR | atgtcacattTTGTGCGTAATTTATTCACAAGCTTGCATTGAACTTGTGGataaaatcac |
| CytR | atcacttttgGTGGGTAAATTTATGCAACGCATTTGCGTCATGGTGATGAgtatcacgaa |
| CytR | ttcaatattcATCACACTTTTCATGAAAATTCTGTAACCGTTTTCACGCGctatctgcta |
| CytR | cgcattcattTGAAAGTGAATTATTTGAACCAGATCGCATTACAGTGATGcaaacttgta |
| CytR | ttgaaccagaTCGCATTACAGTGATGCAAACTTGTAAGTAGATTTCCTTAattgtgatgt |
| IHF | atttaatttaTTGATTATAAAGGgctttaattt |
| IHF | gtataccagtAAAACTATTCATTcgcggaatct |
| IHF | agattccgcgAATGAATAGTTTTactggtatac |
| IHF | aacataaaacACTATCAATAAGTtggagtcatt |
| IHF | aacataaaacACTATCAATAAGTtggagtcatt |
| IHF | caccatcataCACTAAATCAGTAagttggcagc |
| IHF | ataaattggcTAATAGATTTATTtttattcagc |
| IHF | tttaatttttAATCATCTAATTTgacaatcatt |
| IHF | tttgccgcatCTCGAAAAATCAAggagttgcaa |
| IHF | tacgctaaatAATCACTGTGTTGagtgcacaat |
| IHF | tatatctaacTCATTGAATCTTTattagttttg |
| IHF | tctctttgatTTTCAAATTATTCgatgtataca |
| IHF | tttttatttgGATAATCAAATATttactccgta |
| IHF | cgttgaaggtTACCTGCGTGCTTctgaagcatc |
| IHF | cgtgcttctgAAGCATCCCGTGAccgcgttgaa |
| IHF | aaaaccgcgcAATCAGCCTGTCTgttcgtgcga |
| IHF | tgcaatcgcaACTGTTAACAAACaggaagatgc |
| IHF | taaaggcgagTAATTCTCTGACTcttcgggatt |
| IHF | ttcgtcctgtAATCAAGCACTAAgggcggctac |
| IHF | ttctttttgaAACCAAATCTTTAtctttgtagc |
| IHF | ttttctttttGAAACCAAATCTTtatctttgta |
| IHF | cggtgttcacAAAGTTCCTTAAAttttactttt |
| IHF | taaggaaatcATATAAATAGATTaaaattgctg |
| IHF | aatataaggaAATCATATAAATAgattaaaatt |
| IHF | catcatacacTAAATCAGTAAGTtggcagcatc |
| IHF | cttttcagcaACAAAACTTGATTaacatcaatt |
| IHF | accaatgcacCATTCATGTTATTctcaatagcg |
| IHF | ttcattgctgTAACCTGTTGTTAattaagagct |
| IHF | ttaatttacaGTCTGTTATGTGGtggctgttaa |
| IHF | atcagatcttTATAAATCAAAAAgataaaaaat |
| IHF | gcgatctgtaTAGCAACTGCGGAaaacattaat |
| IHF | attaatgtttTCCGCAGTTGCTAtacagatcgc |
| IHF | gctaaaacttTACCCTGTTGTTAcggcaacagg |
| IHF | agagcctcgcCATAAGCCTGATCctgcaagata |
| IHF | agttggcgtaAATCAGGTAGTTGgcgtaaactt |
| IHF | gcggatatttTATCCGAATGTAAgaaagttggc |
| IHF | caggcttatcTAACACGCTGATAaacaagagga |
| IHF | aacataaaacACTATCAATAAGTtggagtcatt |
| IHF | gctgcatgacAAAGTCATCGGGCattatctgaa |
| IHF | aacccaatacGTAATCAACGACTtgcaatatag |
| IHF | aatgttttatCAAGCGTCTGATTacccgctaca |
| IHF | tagtgatgccTAATAAATCTATTgccggatgtt |
| IHF | agaataagttATTGATTTTAACCttgaattatt |
| IHF | agaataagttATTGATTTTAACCttgaattatt |
| IHF | ttgttaaagtTGACAAAAGGTTAtagaaaggag |
| IHF | agaaacttttGTTAAAGTTGACAaaaggttata |
| IHF | ctaatgtgaaGAAACTTTTGTTAaagttgacaa |
| IHF | tgtcaactatTGAAATGAAAAGTaaaacaattc |
| IHF | agaaagcacaTTCGAAAAAGCGAtaagttatga |
| IHF | tgccaactttTAATTTATTGTTAttaaagagat |
| IHF | atctctttaaTAACAATAAATTAaaagttggca |
| IHF | tttaaacacaAATCTAATTCCTTgatttaaaat |
| IHF | actttaacttGTTGATATTTAAAggtatttaat |
| IHF | ttttctaataTGATGATTTTTATgagtaattat |
| IHF | tttctaatatGATGATTTTTATGagtaattatc |
| IHF | ttctaatatgATGATTTTTATGAgtaattatcg |
| IHF | taagaaaattTATACAAATCAGCaatataccca |
| IHF | acttaagctgCTGTTTAATATGCtttgtaacaa |
| IHF | atcatatcaaCAGAATCAATAATgtttcgccga |
| IHF | aggggcgtttTCATCTCGTTGATtccctttgtc |
| IHF | aggcctacgaAAATTCTGCAATGtattgaattt |
| IHF | aatctgattgATGAATTTCAAAAgacataaaaa |
| IHF | tttatgtcttTTGAAATTCATCAatcagattgc |
| IHF | gtagccaacaAACAATGCTTTATgaatcctccc |
| IHF | gaggaagggaACAACATTCATACtgaaattgaa |
| IHF | ggaagggaacAACATTCATACTGaaattgaatt |
| IHF | tattttatttTTAAAAAACAACAatttatattg |
| IHF | ttttatttttAAAAAACAACAATttatattgaa |
| IHF | tttatttttaAAAAACAACAATTtatattgaaa |
| IHF | cattttcctgCAAAACCATACCCttacgaaaag |
| IHF | cccttacgaaAAGTACGGCATTGataatcattt |
| IHF | taatcattttCAATATCATTTAAttaactataa |
| IHF | ttaactataaTGAACCAACTGCTtacgcggcat |
| IHF | tcgttaagcgATTCAGCACCTTAcctcaggcac |
| IHF | ttcatattgtTATCAACAAGTTAtcaagtattt |
| IHF | catcatacacTAAATCAGTAAGTtggcagcatt |
| IHF | gctatatttaTTTCTCGCCGCGGgagtcggctt |
| IHF | taaacgaaaaATTAAAAAGAGAAgaggtttgat |
| IHF | tcaatttcccTTCCTTATTAGCCgcttacggaa |
| CRP | tgatgacataAGCAGGATTTAGCTCACACttatcgacgg |
| CRP | cgaaacaaaaATGTGATACCAATCACAGAatacagctta |
| CRP | actttcaataTTGGTGATCCATAAAACAAtattgaaaat |
| CRP | gttttcaataTTGGTGAGGAACTTAACAAtattgaaagt |
| CRP | cacattgattATTTGCACGGCGTCACACTttgctatgcc |
| CRP | atagcaaagtGTGACGCCGTGCAAATAATcaatgtggac |
| CRP | atgttgctttTTTGTAAACAGATTAACACctcgtcaaaa |
| CRP | aaagctcgaaTTGTGATGACGATCACACAtgttaaaacc |
| CRP | ggttttaacaTGTGTGATCGTCATCACAAttcgagcttt |
| CRP | acgcaattaaTGTGAGTTAGCTCACTCATtaggcacccc |
| CRP | tgctcattgtTCTGCATATTAATTGACATttctatagtt |
| CRP | attggtagccGTTCTGGTGGTTGTGATGGtggtatgaaa |
| CRP | gcaaactggaAAGTACGTTTGCAGTGAAAtaactattca |
| CRP | aaatgatagaACTGTGAAACGAAACATATttttgtgagc |
| CRP | aatgtgtgtaAACGTGAACGCAATCGATTacgtaaatga |
| CRP | ttttgcaacaTATGTGACCTGGCAGCCAAatccaagtaa |
| CRP | tcatcacccaGTTGTCACTCTAATGATAAttatttgtta |
| CRP | tgactgcaaaATAGTGACCTCGCGCAAAAtgcactaata |
| CRP | caaaacttacATCTTGAAATAATCACATTgattagatga |
| CRP | cagataaaaaTAGAGATCTACTTCACAAAtcaaacgaga |
| CRP | cagataaaaaTAGAGATCTACTTCACAAAtcaaacgaga |
| CRP | gggtttaagcTGTGTGACGAAGTAACCACtcttaacagc |
| CRP | ttcaaaaaatCAGGTCGGATTGACGCCTGtctgcgcaaa |
| CRP | ctaaaccagaAAACTTATTTTATCATTCAaaaaatcagg |
| CRP | tatttggtgaCAAAACTCACAAAAGACACgcgtttaatt |
| CRP | gacaaaactcACAAAAGACACGCGTTTAAtttgcgatac |
| CRP | gatacgaattAAATTTTCACACACTCTGTagcagatgat |
| CRP | gaaatcaaaaAGTAATCTGCTTTATGCCTgatgcgacgc |
| CRP | tgccgttgtaAATGTAAGCTGTGCCACGTttttattaac |
| CRP | tgccgttgtaAATGTAAGCTGTGCCACGTttttattaac |
| CRP | cttatgtaacAGTGTGGAAGTATTGACCAattcattcgg |
| CRP | atttcacctaAGATTAACTTATGTAACAGtgtggaagta |
| CRP | attcaacaaaGTTGTTACAAACATTACCAggaaaagcat |
| CRP | aataaatgttGTTATCGTGACCTGGATCActgttcagga |
| CRP | ttatcctgaaCAGTGATCCAGGTCACGATaacaacattt |
| CRP | gttgttatcgTGACCTGGATCACTGTTCAggataaaacc |
| CRP | tatgggcagcTTCTTCGTCAAATTTATCAtgtggggcat |
| CRP | ggcagcttctTCGTCAAATTTATCATGTGgggcatcctt |
| CRP | gattccactaATTTATTCCATGTCACACTtttcgcatct |
| CRP | gattccactaATTTATTCCATGTCACACTtttcgcatct |
| CRP | gccaggcgagATATGATCTATATCAATTTctcatctata |
| CRP | tatgcctgacGGAGTTCACACTTGTAAGTtttcaactac |
| CRP | gtgcaaccgcAAAAAATGTGAGAGAGTGCaacctgatga |
| CRP | aacctgatgaAAAATAGTGTCGCTGAGCActaaaattta |
| CRP | aatttaatgtAAATGGTGTGTTAAATCGAttgtgaataa |
| CRP | ctgaagttgaAACGTGATAGCCGTCAAACaaattggcac |
| CRP | ttcaaccgttAAATTGATCCCTTTTTAACaaggaatttc |
| CRP | attaaacattGTTTGATATTTATCATATTaatagaaata |
| CRP | gagtcagggaGATGTGAGCCAGCTCACCAtaaaaaagcc |
| CRP | gagtcagggaGATGTGAGCCAGCTCACCAtaaaaaagcc |
| CRP | gagtcagggaGATGTGAGCCAGCTCACCAtaaaaaagcc |
| CRP | ttatcaagatGTGATTAGATTATTATTCTtttactgtat |
| CRP | ttatcaagatGTGATTAGATTATTATTCTtttactgtat |
| CRP | ttatcaagatGTGATTAGATTATTATTCTtttactgtat |
| CRP | cagtgggttaTGTGCGGGCGTGATCACAAttacaaccct |
| CRP | gcctgttgtaAACTGTGAGCCAAAGCGTTgtttaaccaa |
| CRP | cctgttgtaaACTGTGAGCCAAAGCGTTGtttaaccaag |
| CRP | tcttttatcaATTTGGGTTGTTATCAAATcgttacgcga |
| CRP | ataaaaatagGGTGCGAAATCCGTCACAGttcaaacata |
| CRP | tatgtttgaaCTGTGACGGATTTCGCACCctatttttat |
| CRP | ttacacttgtTTTTATGAAGCCCTTCACAgaattgtcct |
| CRP | ggataattatTTTGTGAAGGCTATTAGCCtacacctgta |
| CRP | agcccgaaaaATGTGCTGTTAATCACATGcctaagtaaa |
| CRP | aagcccgaaaAATGTGCTGTTAATCACATgcctaagtaa |
| CRP | attatcgttgCGTAATGTGATTTATGCCTcactaaaatt |
| CRP | tcaaattttaGTGAGGCATAAATCACATTacgcaacgat |
| CRP | atcgttgcgtAATGTGATTTATGCCTCACtaaaatttga |
| CRP | ctgggtcattTTTTTCTTGCTTACCGTCAcattcttgat |
| CRP | gcaaacgaatGTGACAAGGATATTTTACCtttcgaaatt |
| CRP | gaaagttaaaTTACGGATCTTCATCACATaaaataattt |
| CRP | tgagtgttttGTGTGATCTGCATCACGCAttattgaaaa |
| CRP | aattctgcgaTGTGATATTGCTCTCCTATggagaattaa |
| CRP | tgggctaaaaTTTGCGATGCGTCGCGCATttttgatgta |
| CRP | ggctaaaattTGCGATGCGTCGCGCATTTttgatgtatg |
| CRP | gcataattaaTGAGATTCAGATCACATATaaagccacaa |
| CRP | cgctttcaatCTGTGAGTGATTTCACAGTatcttaacaa |
| CRP | gtttccattgCTGTGACTCGATTCACGAAgtcctgtatt |
| CRP | tatttaataaTGTGTGCGGCAATTCACATttaatttatg |
| CRP | aatgttcaaaATGACGCATGAAATCACGTttcactttcg |
| CRP | ttcgaaagtgAAACGTGATTTCATGCGTCattttgaaca |
| CRP | cttcgcgctcCTGTTACAGCACGTAACATagtttgtata |
| CRP | gagatgagctAAAGTGAACCATATCTCAAttcaccttca |
| CRP | tcaccttcatTTTTAGATGTAAATCACTCcattgatgca |
| CRP | acatagcagaAATGTATGACAGATCACTAtttttgaagc |
| CRP | acgtcattatAGTGTGTGTCAGATCTCGTtttccttaac |
| CRP | taggtgctttTTTGTGGCCTGCTTCAAACtttcgcccct |
| CRP | aggtgcttttTTGTGGCCTGCTTCAAACTttcgcccctc |
| CRP | ctgaatcgatTTTATGATTTGGTTCAATTcttcctttag |
| CRP | ctgaatcgatTTTATGATTTGGTTCAATTcttcctttag |
| CRP | gcatacctcaCTTCTCGTGATCAAGATCAcattctcgct |
| CRP | agaaggtaacATGTGAGCGAGATCAAATTctaaatcagc |
| CRP | gctgatttagAATTTGATCTCGCTCACATgttaccttct |
| CRP | caacaagccaACTGCGACCACGGTCACAGcgcctgtaac |
| CRP | agagcaaggaGTTGTGATCAAGCCTGCACaaaattccac |
| CRP | ttactgtgatTAGTTGAACCAGGTCACAAaatataagct |
| CRP | tcaacttaatTTGAAAATTGGAATATCCAtcacataacg |
| CRP | ccccgttaaaAAAATTCTCTTCATTAAATttggtgacat |
| CRP | gccaaccataAATGTGACGGCAATCGATTtaagcgtgac |
| CRP | ggggataaaaGTGTGATGTGAGTCAGATAaatgtcttct |
| CRP | attcttcgttTTGTTACCTGCCTCTAACTttgtagatct |
| CRP | caggggcaaaAATGTTATCCACATCACAAtttcgttttg |
| CRP | tgtttgcaatTATTTGCCACAGGTAACAAaaaaccagtc |
| CRP | tttgcaattaTTTGCCACAGGTAACAAAAaaccagtccg |
| CRP | tccgcatcacGATGTGAGGAAATTAACATgaatcttaag |
| CRP | ttcactaaaaAGTGTGATCGGGGACAATAtatttacgca |
| CRP | gaaagttaatTTGTGAGTGGTCGCACATAtcctgttcat |
| CRP | gctcaaaatgTTTAAATTAACTTATGTAAcagtcacgca |
| CRP | aaacgctaaaCTTGCGTGACTACACATTCttgagatgtg |
| CRP | taaatgcaatTCTTTGATCCATCTCAGAGgattggtcaa |
| CRP | atcgacacgcTGCGTGACGAAGTTGCCAAatttgtcgtt |
| CRP | cgcgcaacggAAGGCGACCTGGGTCATGCtgaagcgaga |
| CRP | gtctcgcttcAGCATGACCCAGGTCGCCTtccgttgcgc |
| CRP | gtactgcatgTATGCAAAGGACGTCACATtaccgtgcag |
| CRP | actgcacggtAATGTGACGTCCTTTGCATacatgcagta |
| CRP | gcattttaagAGGTGATTTTGATCACGGAataaaaagtg |
| CRP | gcattttaagAGGTGATTTTGATCACGGAataaaaagtg |
| CRP | gcattttaagAGGTGATTTTGATCACGGAataaaaagtg |
| CRP | ttaggtgattTTGTGATCTGTTTAAATGTtttattgcaa |
| CRP | ttaggtgattTTGTGATCTGTTTAAATGTtttattgcaa |
| CRP | ttaggtgattTTGTGATCTGTTTAAATGTtttattgcaa |
| CRP | aatttcattcAGGAATGCGATTCCACTCAcaatattccc |
| CRP | taatagcaacCGTTTCGTGACAGGAATCAcggagttttt |
| CRP | gcacattgggTATAACGTGATCATATCAAcagaatcaat |
| CRP | gcacattgggTATAACGTGATCATATCAAcagaatcaat |
| CRP | gcacattgggTATAACGTGATCATATCAAcagaatcaat |
| CRP | gcacattgggTATAACGTGATCATATCAAcagaatcaat |
| CRP | ccccgcgataATATGACCAACCTCTCATAatttaaattt |
| CRP | cactagctttAAGTGGTTGAGATCACATTtccttgctca |
| CRP | aagatttggaATTGTGACACAGTGCAAATtcagacacat |
| CRP | aaacattttaGAGTGATATGTATAACATTatggcgttta |
| CRP | aaacattttaGAGTGATATGTATAACATTatggcgttta |
| CRP | aaacgccataATGTTATACATATCACTCTaaaatgtttt |
| CRP | cgtcaccgccTTGTCATCTTTCTGACACCttactatctt |
| CRP | tgcattgaacTTGTGGATAAAATCACGGTctgataaaac |
| CRP | tgcattgaacTTGTGGATAAAATCACGGTctgataaaac |
| CRP | tgcattgaacTTGTGGATAAAATCACGGTctgataaaac |
| CRP | acaagcttgcATTGAACTTGTGGATAAAAtcacggtctg |
| CRP | ttcccggtatTTCATCTCTATGTCACATTttgtgcgtaa |
| CRP | ttcccggtatTTCATCTCTATGTCACATTttgtgcgtaa |
| CRP | ttcccggtatTTCATCTCTATGTCACATTttgtgcgtaa |
| CRP | gtgctggtttGTGCGAGCCAGCTCAAACTttttaacctt |
| CRP | tatttttcacAAATTTGAGAGTTGAATCTcaaatcatat |
| CRP | tttcacaaatTTGAGAGTTGAATCTCAAAtcatatcaaa |
| CRP | tctgtcgagaTCGTGAACTACGGCACACTttgcgctacc |
| CRP | ggtagcgcaaAGTGTGCCGTAGTTCACGAtctcgacaga |
| CRP | caaggaagccTGGGATGAAAGTGACATTTgagcagttaa |
| CRP | gcccgtaaatTCGTGATAGCTGTCGTAAAgctgttaccg |
| CRP | cgaaggcataACATGCTGTAGATCACATCaggtgaacgc |
| CRP | aagaaaatatCTTGTGATTCAGATCACAAagattcaaca |
| CRP | tcaaaacaaaAATGTGACACTACTCACATttaaatgcca |
| CRP | acacaagcgtTTTGTGATGAACGTCACGTcaattacctc |
| CRP | cccctatattTATGTGATTGATATCACACaaaaggccgt |
| CRP | accgcgcgaaGCGTGATGCATCTCACCTTttcacttcat |
| CRP | ttcagtacaaAACGTGATCAACCCCTCAAttttcccttg |
| CRP | ttcagtacaaAACGTGATCAACCCCTCAAttttcccttg |
| CRP | tgctccccgaACGATTGTGATTCGATTCAcatttaaaca |
| CRP | gctccccgaaCGATTGTGATTCGATTCACatttaaacaa |
| CRP | aagttaataaCTGCGAGCATGGTCATATTtttatcaata |
| CRP | atgtaaaacgTTTCGAGGTTGATCACATTtccgtaacgt |
| CRP | tgttctgttaAATGTGTTTTGCTCATAGTgtggtagaat |
| CRP | gcaaggtgttAAATTGATCACGTTTTAGAccattttttc |
| CRP | aagggtgattTATGTGATTTGCATCACTTttggtgggta |
| CRP | catttgcgtcATGGTGATGAGTATCACGAaaaaatgtta |
| CRP | gctgtcacgtTTTGTGATGGCTATTAGAAattcctatgc |
| CRP | gcagagtcccTTTGTGATCGCTTTCACGGagcataaaaa |
| CRP | attcagcaaaTTGTGAACATCATCACGTTcatctttccc |
| CRP | gaattgtggtGATGTGATGCTCACCGCATttcctgaaaa |
| CRP | aatgcccgagATGTGAAGCAAATCACCCActtaatgccg |
| CRP | cacacattttAAGTTCGATATTTCTCGTTtttgctcgtt |
| CRP | aacgctcattTTATGACGAGGCACACACAttttaagttc |
| CRP | ggatcgaaaaATTCAATATTCATCACACTtttcatgaaa |
| CRP | ggatcgaaaaATTCAATATTCATCACACTtttcatgaaa |
| CRP | atgaactggaACTGTAAAAGGAAACATCAtggaactgta |
| CRP | atgtaaccgtCAATTTGCGACGCGTCTCAcaagacgctg |
| CRP | aagaccagaaAACGTGATTTAACGCCTGAtttgtcgtac |
| CRP | acatacacaaAAAATATAGATCTCCGTCAcatttttgcg |
| CRP | aaaaaatataGATCTCCGTCACATTTTTGcgttatacag |
| CRP | accgtcgcttTGTGTGATCTCTGTTACAGaattggcggt |
| CRP | accgccaattCTGTAACAGAGATCACACAaagcgacggt |
| CRP | tggcgattttATGTGCGCATCTCCACATTaccgccaatt |
| CRP | aattggcggtAATGTGGAGATGCGCACATaaaatcgcca |
| CRP | cgccacgattTTTGCAAGCAACATCACGAaattccttac |
| CRP | gtaaggaattTCGTGATGTTGCTTGCAAAaatcgtggcg |
| CRP | acgaaatccaTGTGTGAAGTTGATCACAAatttaaacac |
| CRP | gggtgaaaacCGTGCTCCCACTCGCAGTCatcctccctc |
| CRP | ccaatattcaCTGTGAGGTATTTGCTAAAgccggtaacg |
| CRP | atcccaaagcGGTGATCTATTTCACAAATtaataattaa |
| CRP | ccgatcgcccATCGTGCTCGCTTTCACGCgctctaccag |
| CRP | gaatgcgccaACTGTGATAGTGTCATCATtttcaaagcg |
| CRP | atgttgcgctCAGGTGAATCGCGCCAGCAaattacggat |
| CRP | ctgcagccaaCCAAAAATGTCATCTGCCAtggggcttta |
| CRP | tttcctgaacGATAAATTGTGATCTTCGCtgcgtttcgg |
| CRP | caaccattgtTGCGATGAATGTCACATCCtctgatcaat |
| CRP | gagtgaaattCTTGTGATGTGGTTAACCAatttcagaat |
| CRP | cggtttcaaaATTGTGATCTATATTTAACaaagtgatga |
| CRP | ttaacaaagtGATGACATTTCTGACGGCGttaaataccg |
| CRP | gaaagtgaatTATTTGAACCAGATCGCATtacagtgatg |
| CRP | agatttccttAATTGTGATGTGTATCGAAgtgtgttgcg |
| CynR | taattgatatGTCGAGAGAGCATTCGCAACCTATAAGTAAATCCAATGGAACTCATCATAAATGAGACTTttaccttatg |
| CynR | ctcgccgattGTCATAAGGTAAAAGTCTCATTTATGATGAGTTCCATTGGATTTACTTATAGGTTGCGAAtgctctctcg |
| CynR | gagcattcgcAACCTATAAGTAAATCCAATGGAACTCATCATAAATGAGACTTTTACCTTATGACAATCGgcgagtagtc |
| CynR | ggaatgagagGCAGACTACTCGCCGATTGTCATAAGGTAAAAGTCTCATTTATGATGAGTTCCATTGGATttacttatag |
| CynR | ttcaatattgTCATTTTTTCTTCCTCTAATTATATGTAAATCCTATGGATTTTGAATTTAGGGAAGGCGGcaagtttatt |
| NarL | gcaggcgacgTGATGGATCACATTTATCGccaggccatt |
| NarL | gtattcatggTAATGCCACCCAGACCAGCattcctggcg |
| NarL | tgataccgaaCAATAATTACTCCTCACTTacacgtaata |
| NarL | tcctcacttaCACGTAATACTACTTTCGAgtgaaaatct |
| NarL | actttcgagtGAAAATCTACCTATCTCTTtgattttcaa |
| NarL | gagatatgatCTATATCAATTTCTCATCTataatgcttt |
| NarL | ccgtatttgcATAAAAACCATGCGAGTTAcgggcctata |
| NarL | ttttaattaaCTGTTTTAGCGGAGGATGCggaaaaaatt |
| NarL | tgtaattataAGGTTAAATATCGGTAATTtgtatttaat |
| NarL | tttgtgcaaaAGTTTCACTACGCTTTATTaacaatactt |
| NarL | actacgctttATTAACAATACTTTCTGGCgacgtgcgcc |
| NarL | catacccggaAAGAGTTACTCCTTATTTGccgtgtggtt |
| NarL | tttatgaagtCACTGTACTCACTATGGGTaatgataaat |
| NarL | ttaatatgttACCCATGGGGAATACTCCTtaatacccat |
| NarL | ggaatactccTTAATACCCATCTGCATAAaaatcttaat |
| NarL | actccttaatACCCATCTGCATAAAAATCttaatagttt |
| NarL | ggtggctgttAATTATCCTAAAGGGGTATcttaggaatt |
| NarL | ttatcctaaaGGGGTATCTTAGGAATTTActttattttt |
| NarL | ggggtatcttAGGAATTTACTTTATTTTTcatccccatc |
| NarL | ttaggaatttACTTTATTTTTCATCCCCAtcactcttga |
| NarL | tatttttcatCCCCATCACTCTTGATCGTtatcaattcc |
| NarL | cacgtaatcaGTACCCAGAAGTGAGTAATcttgcttacg |
| NarL | gcatttttcaCCTCCTAACTACTTAAAATtgctatcatt |
| NarL | aactggcctgTCAGGCAGTGGTGCGTTTTtctaccgcta |
| NarL | gcagtggtgcGTTTTTCTACCGCTATTGAggtaggtcaa |
| NarL | ggtgcgttttTCTACCGCTATTGAGGTAGgtcaatttgc |
| NarL | ggtgcgttttTCTACCGCTATTGAGGTAGgtcaatttgc |
| NarL | ggtcaatttgCGAAGGCGGATTATTTTGTggcaaacaga |
| NarL | gctacaggttTTACCCCGATCGGGGTATGcatctttgac |
| NarL | gaaagggcatTAAATGCTAATGGTGTTGAtattatgtaa |
| NarL | gataaccataATAAATGTGTGGTAAATGGcgcatcgatc |
| NarL | tcttgctttaATTAATTACACTAATGCTTcttcccttcg |
| NarL | gaaaattgatCAAACATACGTATTATCTTgctttaatta |
| NarL | aaatcagcaaTATACCCATTAAGGAGTATataaaggtga |
| NarL | gaagatactgACTAACTCTAAAGTGGTATtttacatgca |
| NarL | gtattttacaTGCACTTACAATTGATTAAagacaacatt |
| NarL | aagacaacatTCACAGTGTGGTTATTTGTtacacatagg |
| NarL | tatttactatCCAACTTATCATAATACTGatatgtctgt |
| NarL | gattatcgcgAGGGTTCACACATGTTATTtactatccaa |
| NarL | cagcaaaaatTTAAATAGGATTATCGCGAgggttcacac |
| NarL | ctctgaaaacAGTTCATACAAAACAGAACgtgactgtga |
| NarL | cagccaatatTCACTGTGAGGTATTTGCTaaagccggta |
| NarL | ctgttgtaccTATAAAGGAGCAGTGGAATagcgttcgca |
| NarL | tatactgttgTACCTATAAAGGAGCAGTGgaatagcgtt |
| NarL | tcagactataCTGTTGTACCTATAAAGGAgcagtggaat |
